# Progress in Lewis-Acid-Templated Diels–Alder Reactions

**DOI:** 10.3390/molecules29051187

**Published:** 2024-03-06

**Authors:** Jun Ishihara

**Affiliations:** Graduate School of Biomedical Sciences, Nagasaki University, 1-14 Bunkyo-machi, Nagasaki 853-8521, Japan; jishi@nagasaki-u.ac.jp

**Keywords:** Lewis acid, template, Diels–Alder reaction, natural product synthesis

## Abstract

The synthesis of natural products with complicated architectures often requires the use of segments with functional groups that can be structurally transformed with the desired stereogenic centers. Bicyclic 𝛾-lactones have great potential as a suitable segment for natural product synthesis. However, the stereoselective construction of such functionalized bicyclic 𝛾-lactones is not as straightforward as one might expect. The template-mediated Diels–Alder reaction is one of the most powerful and versatile methods for providing bicyclic 𝛾-lactones with high regioselectivity and stereoselectivity. In this reaction, the diene is linked to the dienophile by a temporary tether, allowing the reaction to proceed efficiently, yielding a product that can be used for natural product synthesis. This review describes some important instances of the template-mediated Diels–Alder reaction and its application to the synthesis of biologically active compounds.

## 1. Introduction

Naturally occurring organic compounds are important as drug leads and tools for biological research [1,2,3,4]. However, natural products with promising biological activity can often require structural modification due to insufficient availability in nature, lack of chemical stability, or toxicity. In such cases, it is necessary to generate the desired molecules by means of precise chemical synthesis. Various catalysts for carbon–carbon-bond formation have been developed for the precise and efficient synthesis of natural products [5,6,7,8,9,10,11]. However, these methods are not always effective for substrates with multiple functional groups. Therefore, new catalysts for organic reactions are not always sufficient, and their development continues to be of great importance to the synthetic community.

**Figure 1:**
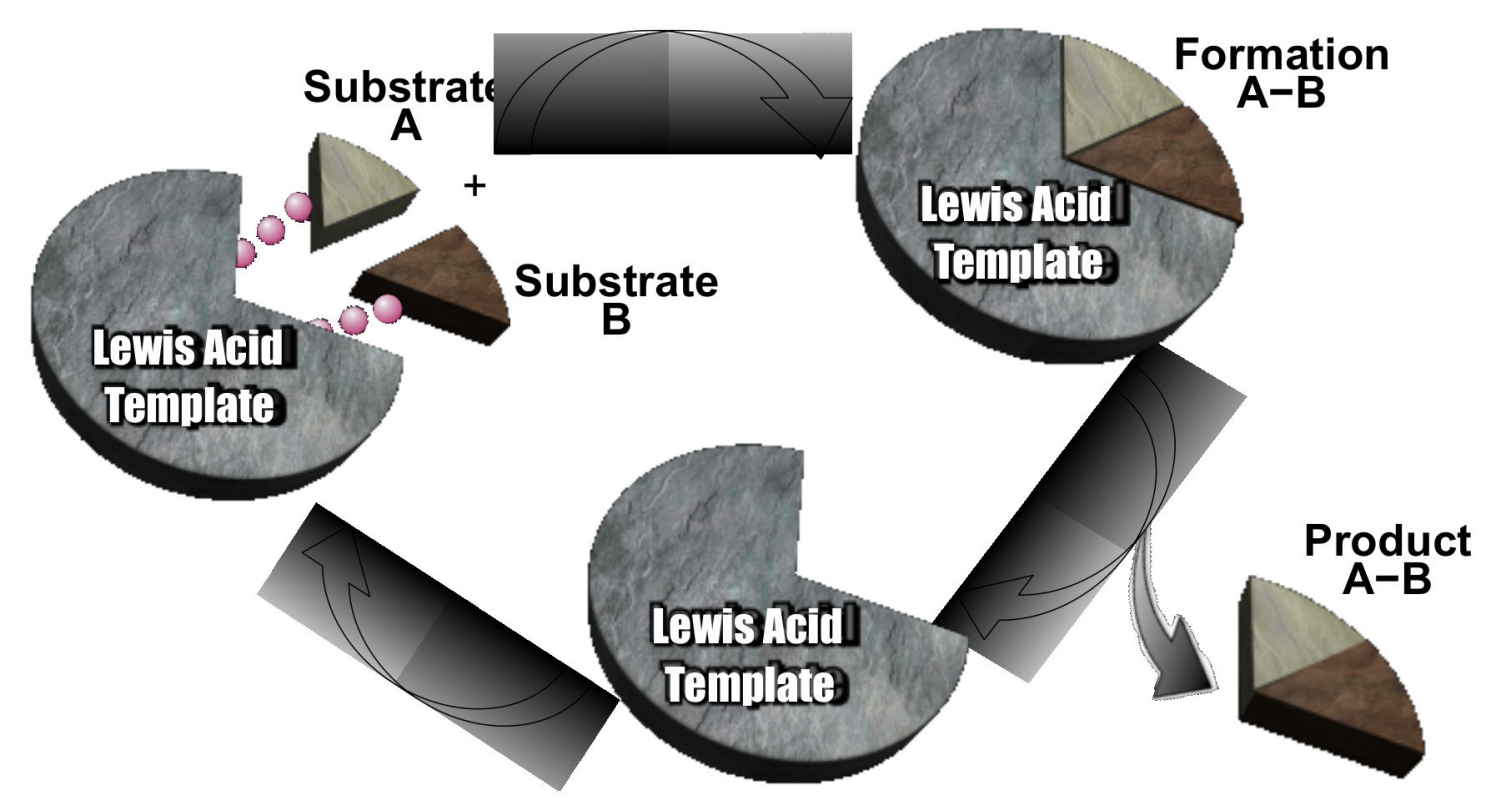
Illustration of a Lewis-acid-templated reaction.

The Diels–Alder reaction is regarded as one of the most important reactions in precise organic synthesis [12,13,14,15,16,17,18,19]. The focus of research on the Diels–Alder reaction has been the development of catalysts that can activate dienophiles. However, catalytic strategies that bring the diene and a dienophile into close proximity and activate the dienophile at the same time considerably increase the efficiency of the cycloaddition. One such strategy involves the use of a Lewis acid template (Figure 1). Substrates A and B meet via Lewis acid and are activated to form a new A–B bond. The newly formed A–B product is released and the catalyst is simultaneously regenerated. In this particular case, the Lewis acid acts as a template that activates the substrates as well as mediates spatial proximity.

This review focuses on such template-mediated Diels–Alder reactions and discusses their research progress to date. Among the wide variety of products that the template-mediated Diels–Alder reaction generates, bicyclic γ-lactones are of particular importance as intermediates in the synthesis of natural products and drug leads. Accordingly, we first describe the preparation of bicyclic γ-lactones and then discuss the templated Diels–Alder reaction as a means of producing them.

## 2. Synthesis of Bicyclic 𝛾-Lactones through the Diels–Alder Reaction

### 2.1. Bicyclic γ-Lactones as Intermediates in Natural Product Synthesis

**Figure 2:**
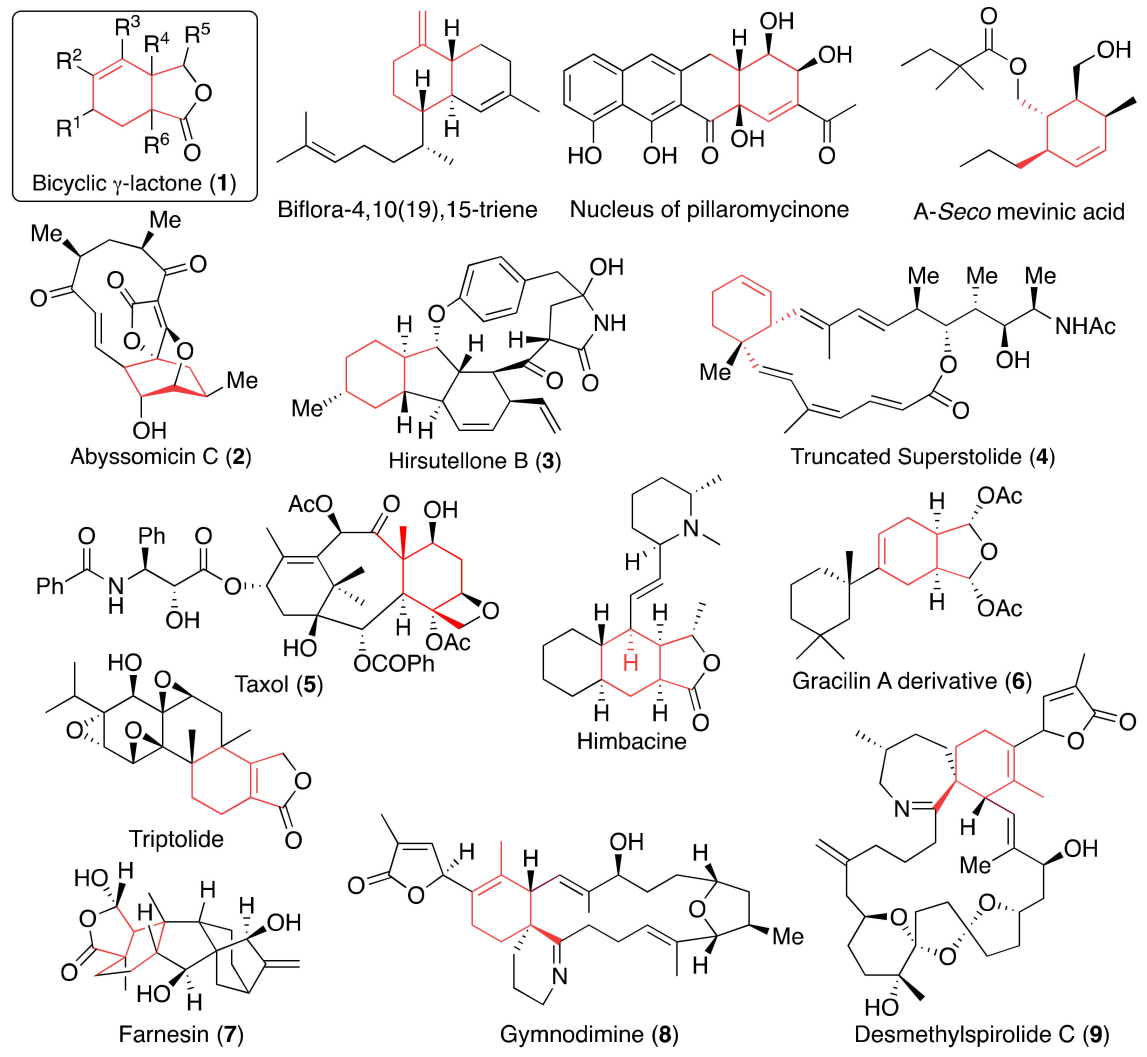
Representative natural products and natural-product-related substances synthesized using a bicyclic 𝛾-lactone as a key intermediate.

The functional groups in the bicyclic 𝛾-lactone shown in Figure 2 (**1**), fused to an unsaturated six-membered ring, can be easily transformed into various substituents through chemical reactions. As a result, such bicyclic molecules have been utilized in the synthesis of many natural products, such as highly functionalized terpenoids and polyketides. Representative syntheses of natural products and natural-product-related substances, using a bicyclic 𝛾-lactone as a key intermediate, are the total synthesis of biflora-4,10(19),15-trien by Grieco [20]; the synthesis of the core of pillaromycinone by White [21,22]; the formal synthesis of A-seco mevinic acid by Potier [23]; the total synthesis of abyssomicin C (**2**) by Nicolaou [24,25,26]; the synthesis of the core of abyssomicin C (**2**) by Maier [27]; the synthesis of the core of hirsutellone B (**3**) by Roush [28]; the synthesis of truncated superstolide (**4**) by Jin [29]; the total synthesis of taxol (**5**) by Nicolaou [30,31,32,33,34]; the synthesis of simplified derivatives of gracilin A (**6**) by Romo [35]; the formal synthesis of triptolide by Sherburn [36]; a synthetic approach to himbacine by Sherburn [37]; a synthetic approach to the spirolide upper fragment by Ishihara [38]; the synthesis of the spirocyclic core of gymnodimime (**8**) and desmethylspirolide C (**9**) by Landais [39]; and the total synthesis of farnesin (**7**) by Gao [40].

### 2.2. Diels–Alder Reactions That Produce Bicyclic γ-Lactones

**Scheme 1:**
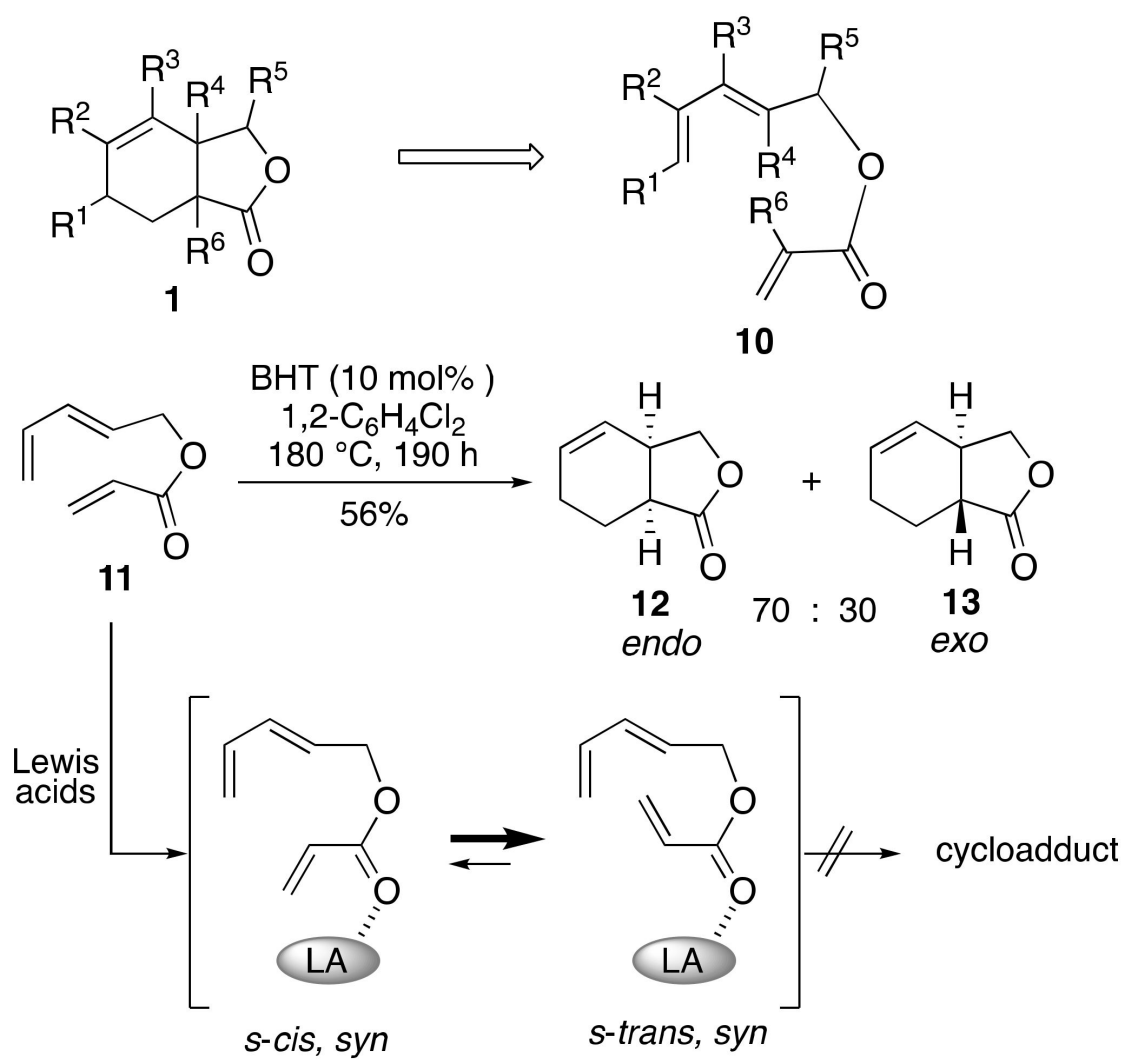
Intramolecular Diels–Alder (IMDA) reaction of pentadienyl acrylate.

As shown in Scheme 1, functionalized bicyclic 𝛾-lactones (**1**) are readily obtained by the intramolecular Diels–Alder (IMDA) reaction of the corresponding pentadienyl acrylate (**10**). However, their stereoselective construction is not as straightforward as one might expect. This is because the sp^2^ carbon of the internal carbonyl group leads to steric distortion during cyclization of the triene ester. The steric distortion of intramolecular reactions is more dominant than that of secondary orbital interactions, making the general *endo* rule less relevant. Accordingly, the thermal IMDA reaction of 2,4-pentadienyl acrylate **11** gives a mixture of the *cis-* and *trans*-fused adducts **12** and **13** in moderate yields [41,42]. Furthermore, the activation of such pentadienyl acrylates with Lewis acids does not promote the Diels–Alder reaction efficiently. This is because when the Lewis acid coordinates to the carbonyl group, *s*-*trans*-*syn* coordination is preferred to *s*-*cis*-*syn* coordination. This coordination makes it virtually impossible for the diene and dienophile connected by a three-atom chain to adopt a conformation that is suitable for the intramolecular cycloaddition [43]. Therefore, the construction of such bicyclic lactones via Diels–Alder reactions with Lewis acids requires ingenuity.

**Scheme 2:**
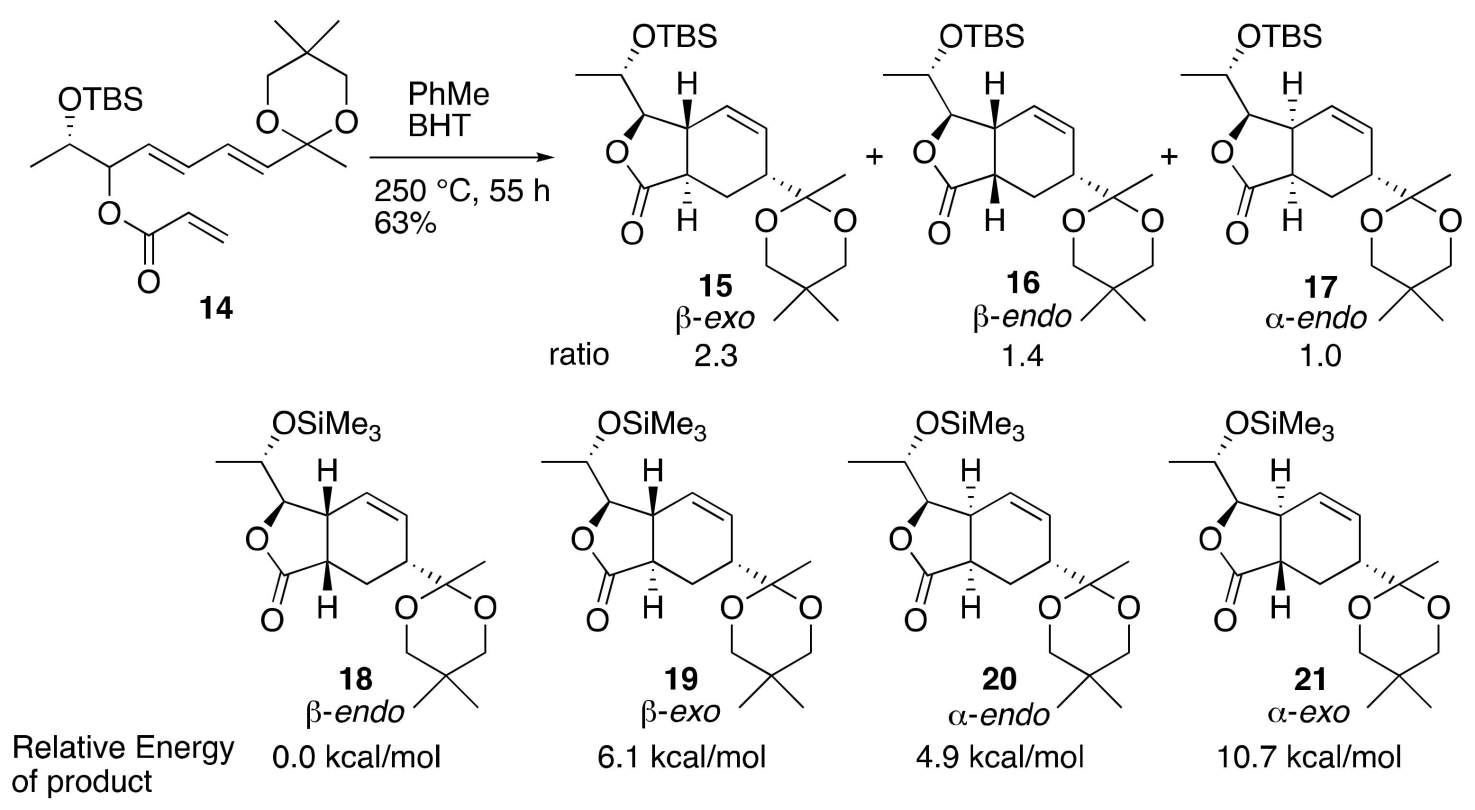
Conformational study results for an IMDA reaction by White et al. [44].

There are many examples of the IMDA reaction being used for the formation of 𝛾-lactones and some are shown here. White et al. reported IMDA reactions of complicated substrates (Scheme 2) [44]. In this case, the IMDA reaction of pentadienyl acrylate **14** at 250 °C gave a mixture of three cycloadducts, **15**, **16**, and **17**, in the ratio 2.3:1.4:1.0 in a 63% overall yield. They also performed a conformational analysis of the IMDA reaction with a simplified model substrate, reporting that the product ratios were in close agreement with those obtained by combined force field and density functional theory (DFT) calculations. Their energy calculations for the MM2* conformational global minima of the four cycloadducts showed that the β-*endo* cycloadduct was the most stable. Apart from the relative energy of the products, the transition states of each reaction were calculated by DFT at the Becke3LYP level, whereby the relative Boltzmann populations of which showed that the relative transition state energies for β-*exo* (**19**), β-*endo* (**18**), α-*endo* (**20**), and α-*exo* (**21**) at 250 °C were 2.3:0.2:0.2:0.06. The calculated results therefore show that when the reaction is under kinetic control, the β-*exo trans*-fused isomer (**19**) is the preferred product, despite its lower stability.

## 3. Selective IMDA Reactions of Pentadienyl Acrylates

### 3.1. Stereo-Directing Strategy

**Scheme 3:**
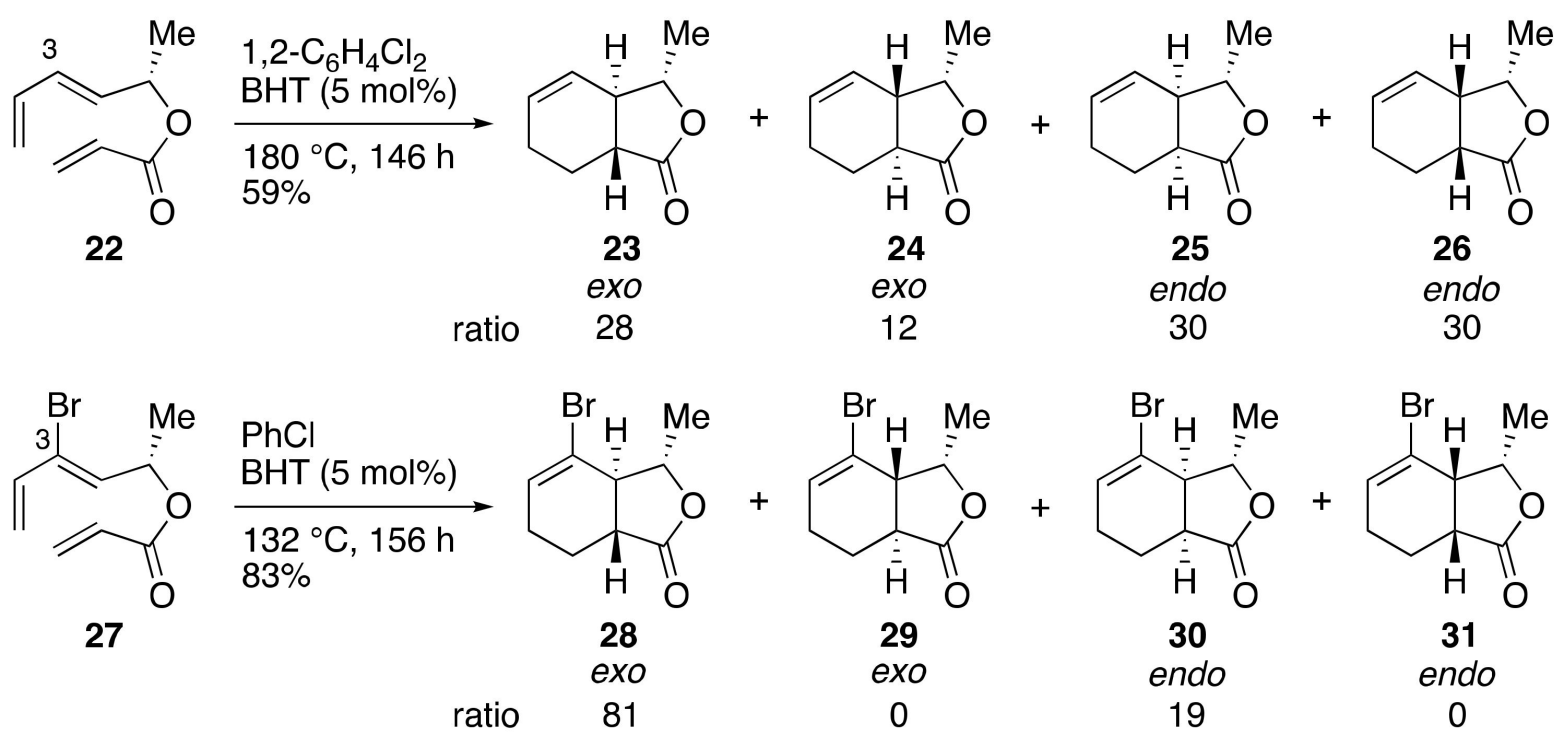
Conformational study results for IMDA reactions by Paddon-Row and Sherburn et al. [41].

The functional group at C3 is known to have a significant influence on the stereochemical outcome of IMDA reactions. This influence has received considerable attention since Boeckman [45] and Roush [46,47] independently developed its stereo-directing effects. The stereo-directing group approach to the formation of bicyclic 𝛾-lactones has been demonstrated by Paddon-Row and Sherburn et al. (Scheme 3) [41]. The IMDA reaction of 5-methyl pentadienyl acrylate (**22**) afforded four isomers (**23**–**26**) in a 59% overall yield, with three of the isomers being produced in similar relative amounts. In stark construct, the thermal IMDA reaction of the substrate brominated at C3 proceeded more smoothly than the corresponding des-bromo substrate to provide two Diels–Alder cycloadducts in an 83% overall yield at a ratio of 81:19 (**28**, **30**). Paddon-Row and Sherburn et al., also described the stereochemical outcome of these reactions in their DFT-calculated analysis.

### 3.2. Diels–Alder/Lactonization Cascade Strategy

**Scheme 4:**
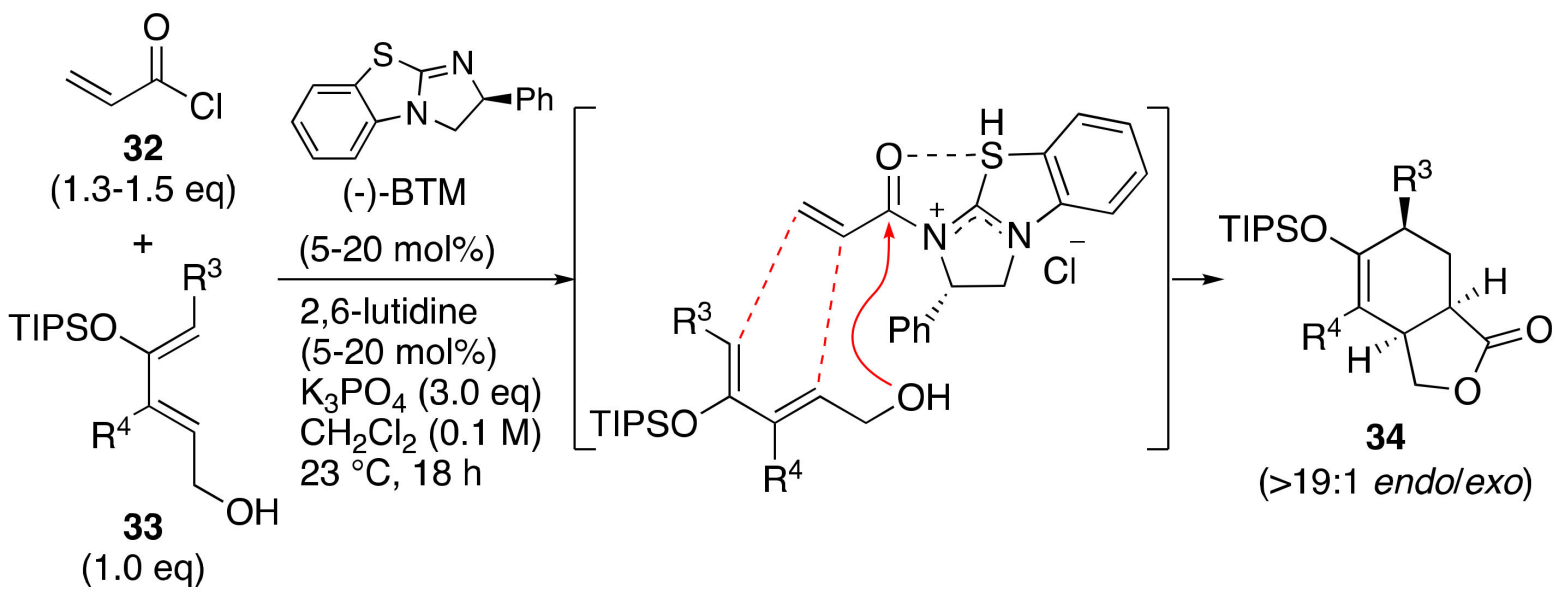
DAL cascade strategy by Romo.

Romo and co-workers have described the potential of α,β-unsaturated acylammonium salts as catalysts for the Diels–Alder cycloaddition reaction (Scheme 4) [48]. α,β-unsaturated acyl ammonium salt was prepared in situ from an acid chloride (**32**) and chiral isothiourea organocatalyst (-)-BTM and it promoted Diels–Alder reactions with dienes (**33**) and subsequent lactonization to produce *cis*- and *trans*-fused bicyclic γ-lactones (**34**) in a good yield with high diastereo- and enantioselectivity. The single operation strategy, termed a Diels–Alder/lactonization (DAL) cascade, was applicable for a variety of bicyclic lactones. (In this review, carbon–carbon formation via the Diels–Alder reaction is often highlighted in red.)

**Scheme 5:**
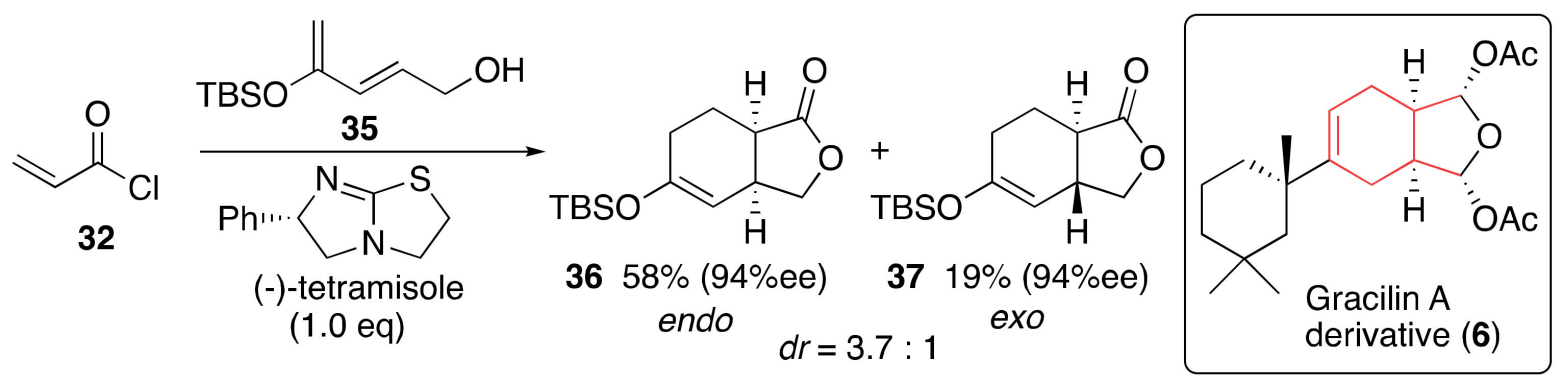
Romo’s synthesis of the gracilin A derivative core.

More recently, Romo and co-workers used the DAL cascade strategy to construct the pharmacophore of gracilin A, which is a promising lead compound for both immunosuppression and neuroprotection (Scheme 5) [35]. Using (-)-tetamisole as the Lewis base promoter, the DAL cascade afforded the required *endo*-diastereomer (**36**) in a 58% yield with 94% ee. The cycloadduct was converted to various derivatives to investigate the structure–activity relationship (SAR) profile of gracilin A.

## 4. Template Effects in the Diels–Alder Reaction

In intermolecular Diels–Alder reactions, regioselectivity, diastereoselectivity, and enantioselectivity, as well as *endo* selectivity, can be problematic, whereas IMDA reactions are expected to be less affected by regio- and diastereoselectivity problems. For these reasons, attempts to convert intermolecular Diels–Alder reactions to IMDA reactions by temporarily tethering the diene and dienophile through suitable atoms or groups have been made [5]. Early attempts at templated Diels–Alder reactions have used atoms such as boron, silicon, magnesium, and aluminum for tethering. In these cases, it was hoped that bringing the diene and dienophile closer together would improve reactivity and control regioselectivity. Several templated reactions are discussed below.

### 4.1. Silicon-Tethered Diels–Alder Reactions

**Scheme 6:**
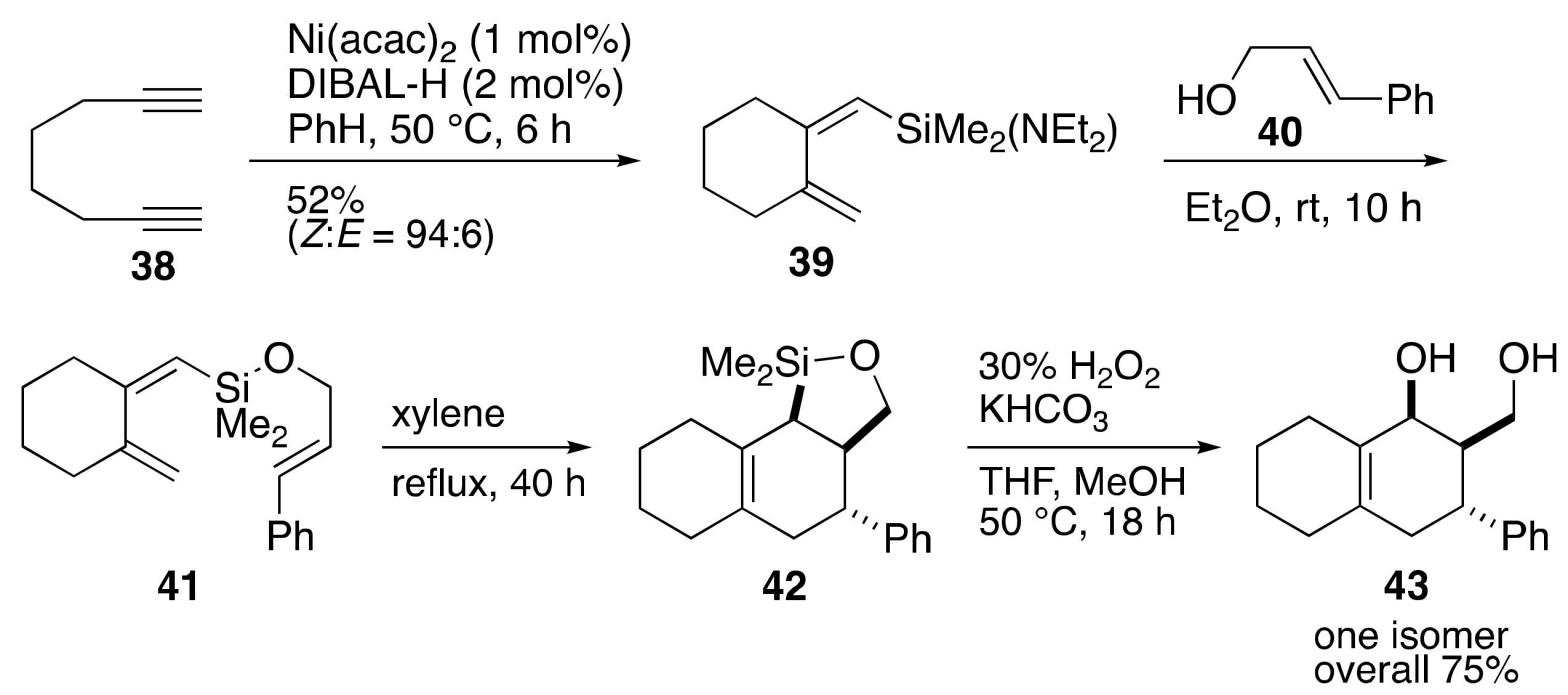
Tamao–Ito’s pioneering IMDA reaction of dienylsilane.

Since the pioneering report by Tamao and Ito, silicon has been widely used as a template to mediate the Diels–Alder reaction (Scheme 6). In their 1989 study on the cyclization of 1,7-diyne (**38**) to 1,2-dialkylidenecyclohexane using nickel(0)-catalyzed hydrosilation with a (Z)-vinylsilane moiety, Tamao, Ito, and their co-workers performed a silicon-templated Diels–Alder reaction [49]. They prepared dienylsilane **39** using a nickel catalyst, followed by coupling with allylic alcohol **40** to give silicon-templated compound **41**. The thermal Diels–Alder reaction of compound **41** proceeded in a highly stereoselective manner, yielding the bicyclic product **43** in an overall yield of 75% with high efficiency via Tamao oxidation.

**Scheme 7:**
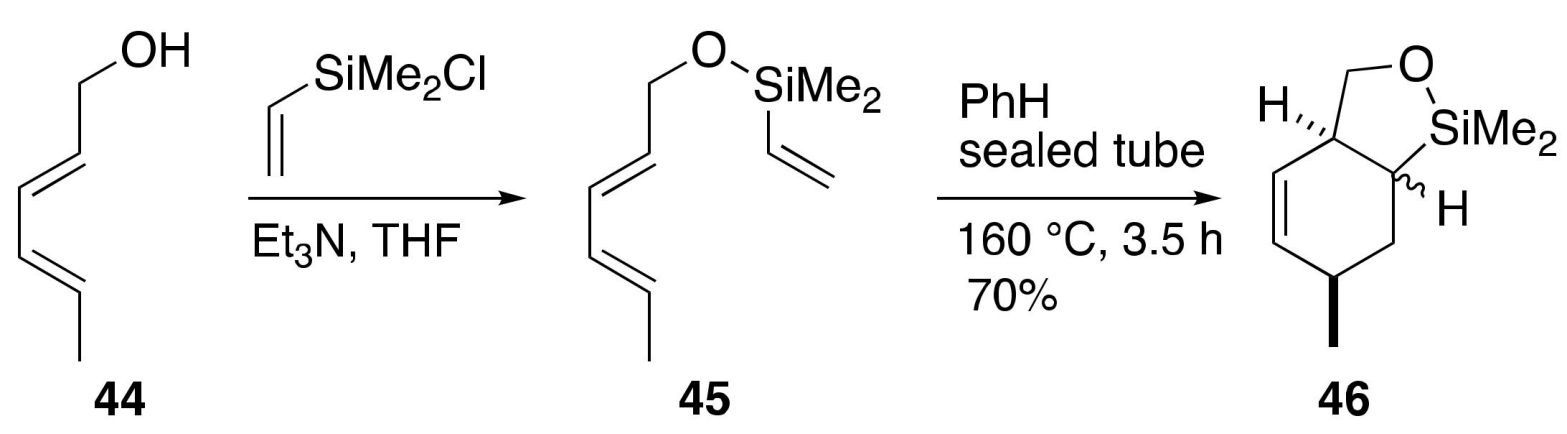
Stork’s silicon-tethered IMDA reaction.

Around the same time, Stork and co-workers carried out a comprehensive investigation of the reaction of silicon-templated substrates using a different geometric isomer (Scheme 7) [50]. They found that the reaction of substituted alkenyl alkenylsilane (**45**) at 160 °C in a sealed tube yielded cyclohexene derivative (**46**) in a 70% yield. The product could be further derivatized by treating with TBAF or by Tamao oxidation. In this case, the functional group’s position on the cyclohexene was fully controlled, demonstrating the usefulness of the templated Diels–Alder reaction. Similar silyl-templated reactions were also reported by Sieburth and co-workers [51].

**Scheme 8:**
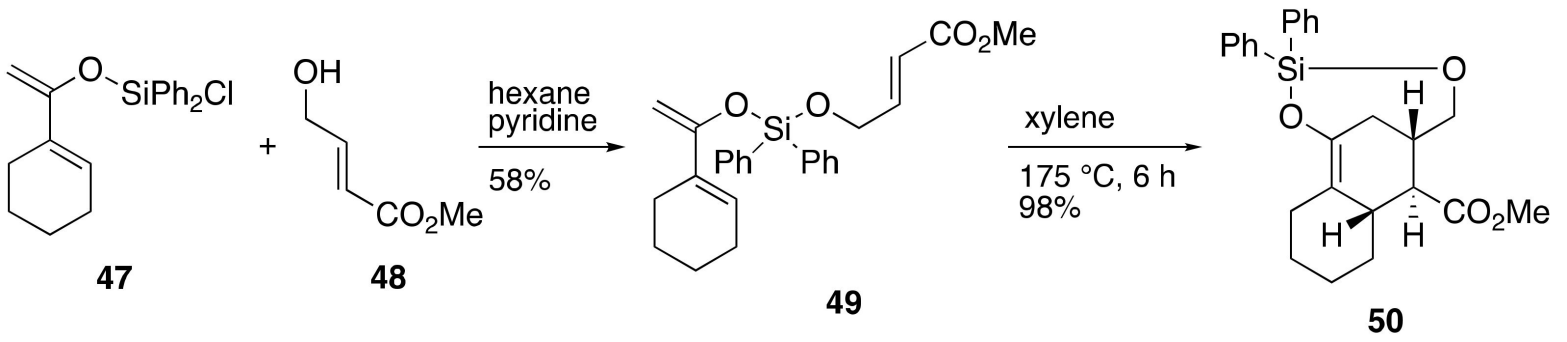
IMDA reaction of dialkoxysilanes by Shea.

Shea et al. have reported Diels–Alder reactions of dialkoxysilanes under thermal conditions (Scheme 8) [52]. In their series of studies, cycloaddition reactions of a number of temporary linkages between dienes and dienophiles using stereochemical constraints were investigated. For example, during the IMDA reaction of diphenylsilyl dienol ether with a methyl acrylate moiety (**49**), the thermal reaction at 175 °C gave a single adduct (**50**) in an excellent yield [11,12].

**Scheme 9:**
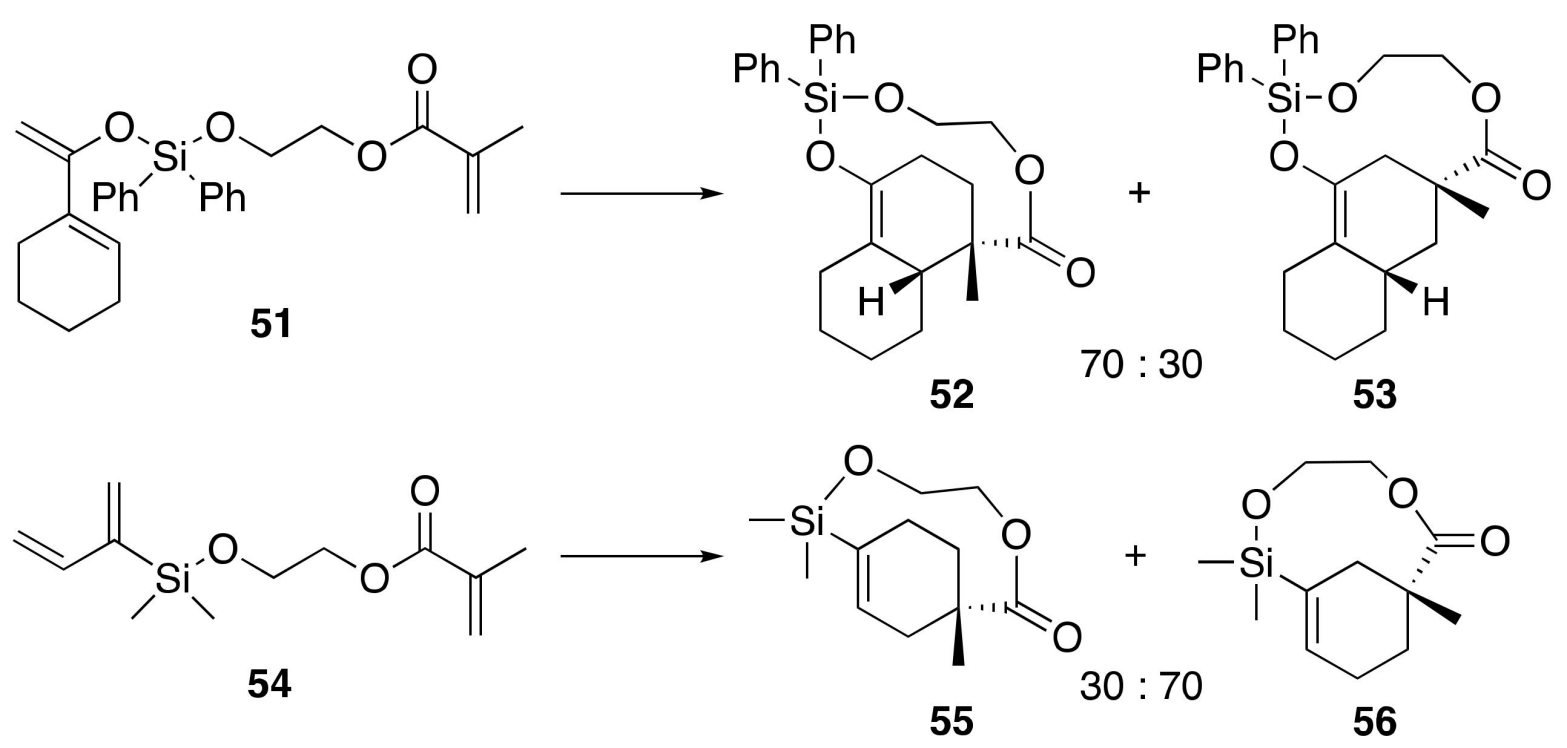
Effect of tether length by Shea.

Shea and co-workers also found that the length of the tether had an effect on the regioselectivity of the reaction (although the reaction temperatures and yields were not reported) (Scheme 9) [53]. The cycloaddition of siloxydiene (**51**) gave a 70:30 mixture of 1,4- and 1,3-regioisomers. In contrast, a 30:70 mixture of 1,4- and 1,3-regioisomers was obtained from the reaction of a substrate with the one-atom-shorter tether (**54**). They concluded that complete regio- and stereochemical control was achieved by shortening the tether to five atoms.

**Scheme 10:**
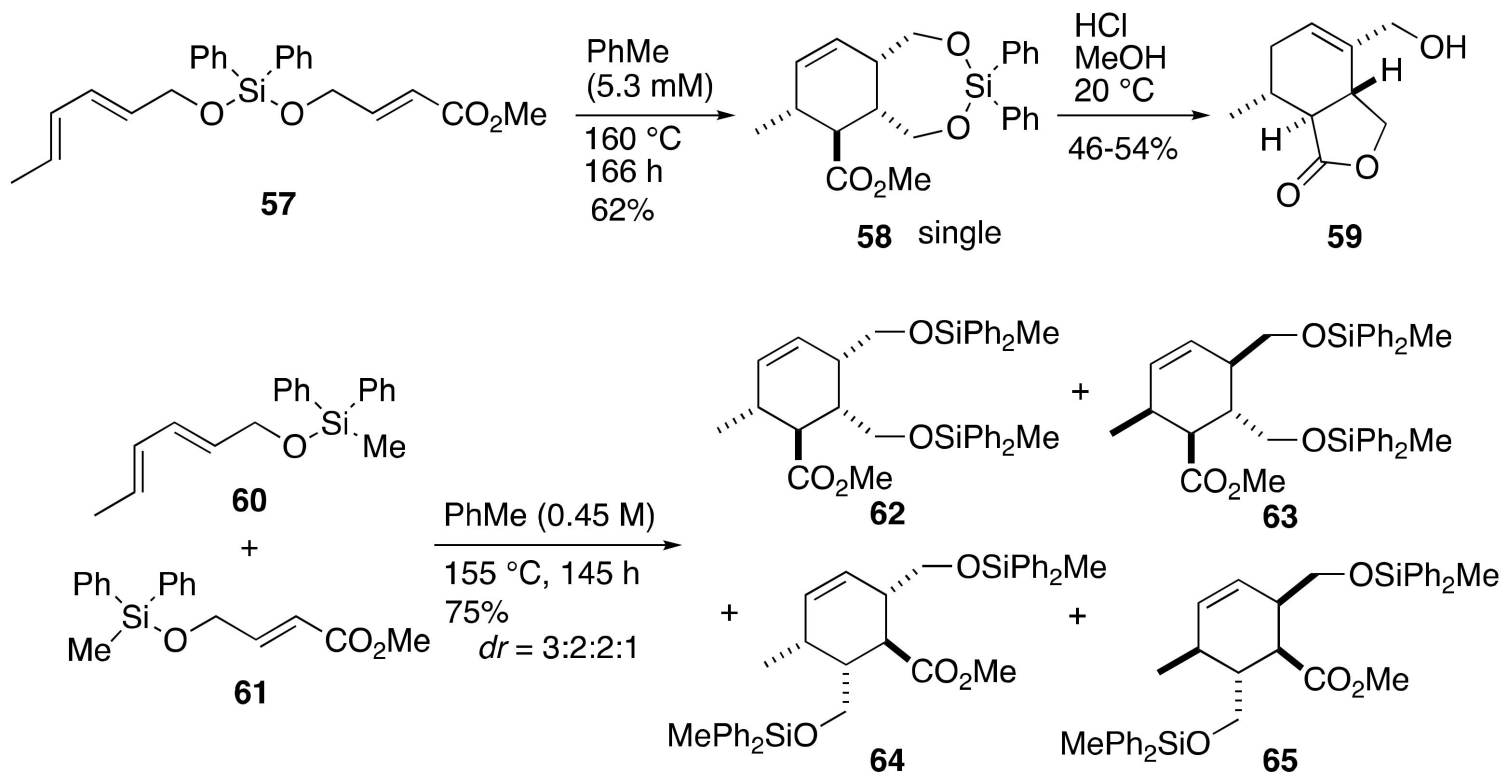
Intramolecular and intermolecular Diels–Alder reactions by Craig.

Other efforts to form bicyclic 𝛾-lactones have been demonstrated by Craig and co-workers (Scheme 10) [54]. The IMDA cycloaddition of a silyl acetal bearing 2,4-hexadienol and methyl 4-hydroxy-2-butenoate was carried out at 160 °C to generate the Diels–Alder adduct (**58**) as a single stereoisomer. The adduct was subjected to acidic conditions to afford bicyclic 𝛾-lactone **59** in a moderated yield. In stark contrast, heating a 1:1 mixture of silyl ethers (**60**, **61**) at 155 °C afforded four adducts corresponding to all possible regio- and stereoisomers (**62**, **63**, **64**, and **65**). These results demonstrate that the simple method of tethering the diene and dienophile components together can significantly influence the regio- and stereochemical outcome of a Diels–Alder reaction.

**Scheme 11:**
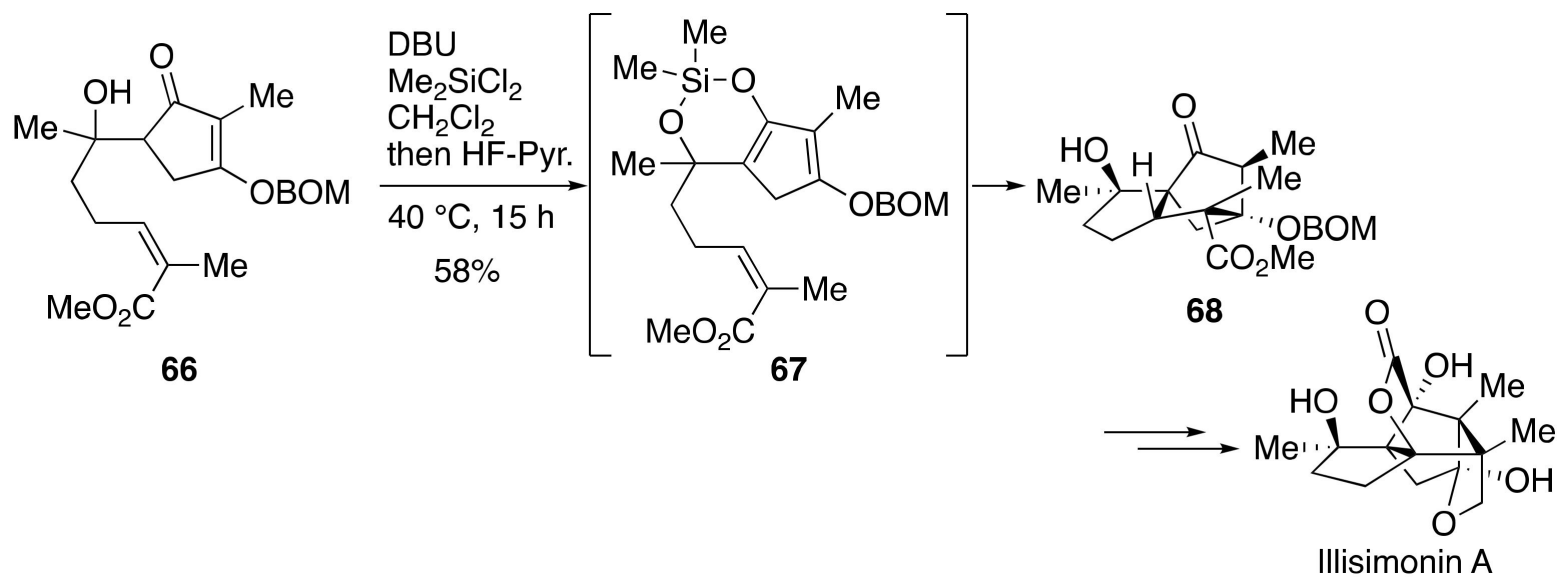
Rychnovsky’s total synthesis of illisimonin A via the silacycle-templated IMDA reaction.

More recently, Rychnovsky and co-workers have developed an IMDA reaction using a silacycle template [55]. In this particular case, a dioxasiline ring serves the dual function of forming a reactive diene and acting as an effective directing group to control the π-facial selectivity of Diels–Alder cyclization. Furthermore, they achieved the total synthesis of (-)-illisimonin A, a neuroprotective sesquiterpenoid, based on the seminal strategy via the silacycle-templated IMDA reaction [56] (Scheme 11). The reaction of dialkoxysilanes has also been studied by Fortin [57] and review articles are available [58,59].

### 4.2. Boron-Templated Diels–Alder Reactions

**Scheme 12:**
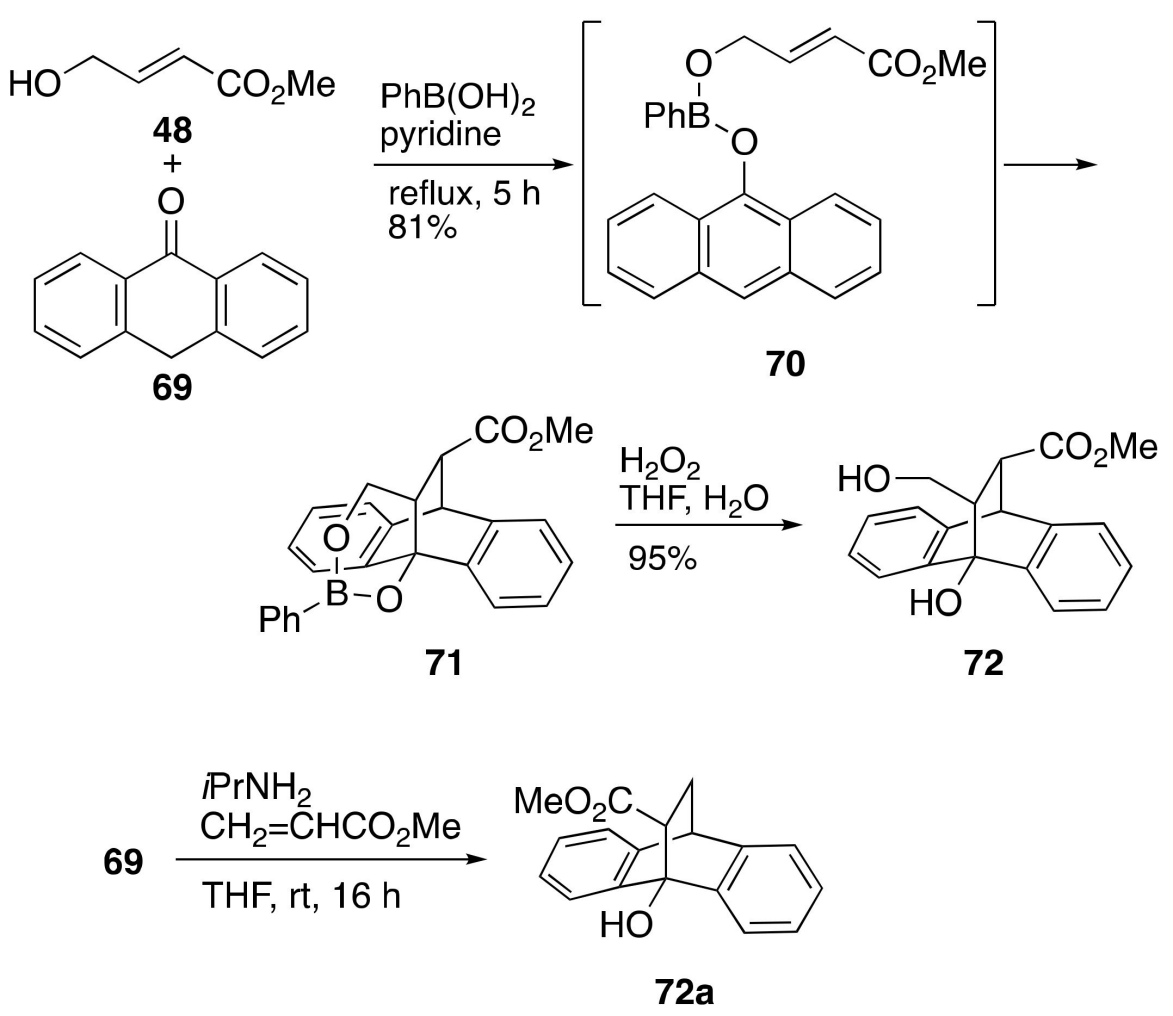
Phenylboronic-acid-templated Diels–Alder reaction by Narasaka and Iwasawa.

Narasaka and Iwasawa et al. reported boron-templated Diels–Alder reactions. They investigated the Diels–Alder reaction of anthrone (**69**) and methyl 4-hydroxy-2-butenoate (**48**) in the presence of phenylboronic acid (Scheme 12) [60]. In this reaction, an enol-type boronic ester was prepared by dehydration and Diels–Alder adduct **71** was formed in a high yield. When anthrone was subjected to the base-catalyzed Diels–Alder reaction of methyl acrylate without phenylboronic acid, the opposite regioisomer (**72a**) was obtained [61], indicating that the phenylboronic acid template completely controls the regioselectivity.

**Scheme 13:**
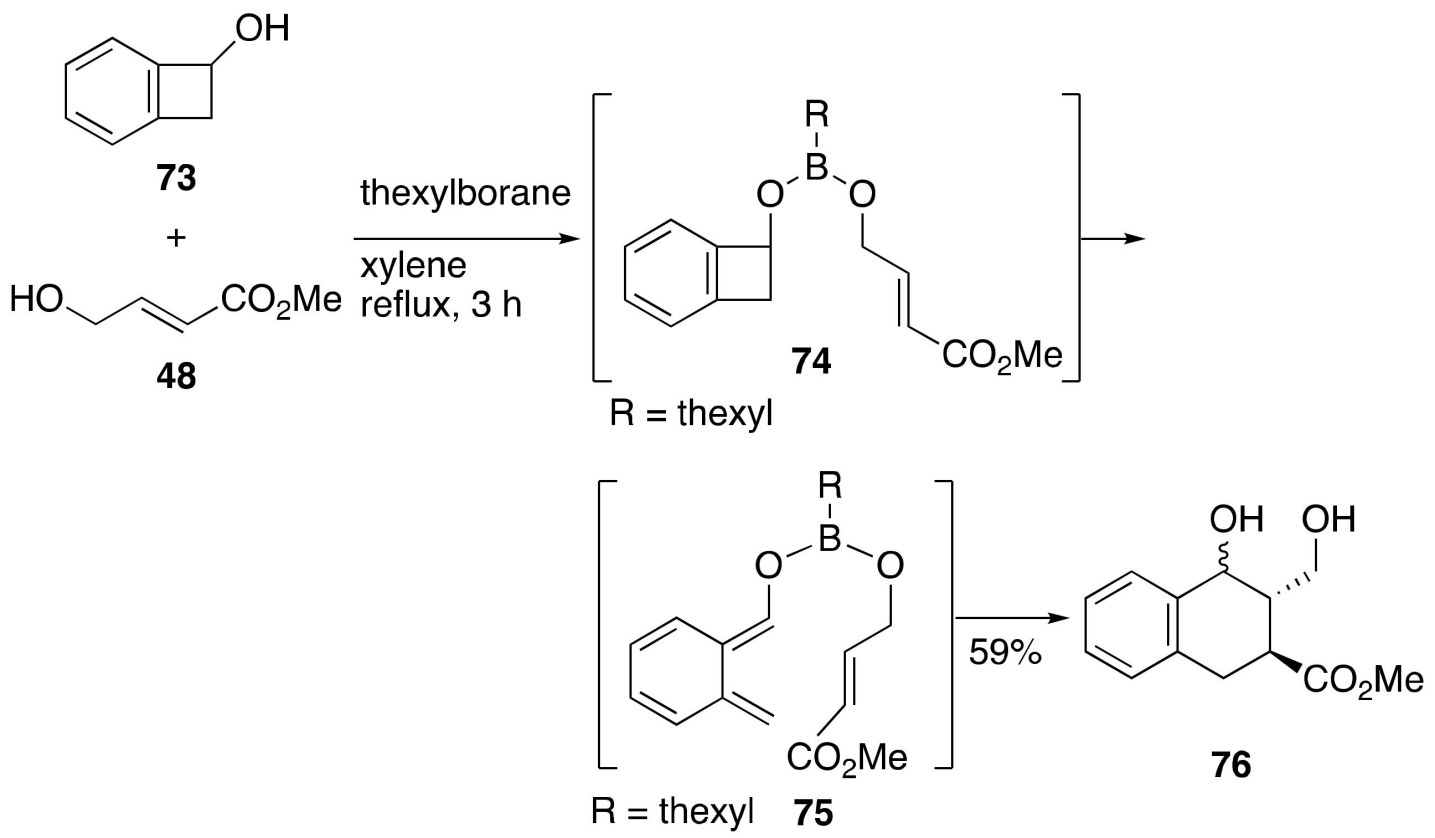
Narasaka’s boron template Diels–Alder reaction.

Narasaka and co-workers also demonstrated a regioselective Diels–Alder reaction controlled by a boronic acid template (Scheme 13) [62]. It is known that 1,2-dihydrobenzocyclobutene derivatives undergo thermal ring opening to generate quinodimethanes. A mixture of 1,2-dihydrobenzocyclobuten-1-ol (**73**) and methyl 4-hydroxy-2-butenoate (**48**) in the presence of thexylborane was stirred to generate a mixed boronate (**74**). Subsequent thermal ring opening, Diels–Alder reaction, and hydrolysis of the resulting boronate afforded cycloadduct (**76**) in a 59% yield.

**Scheme 14:**
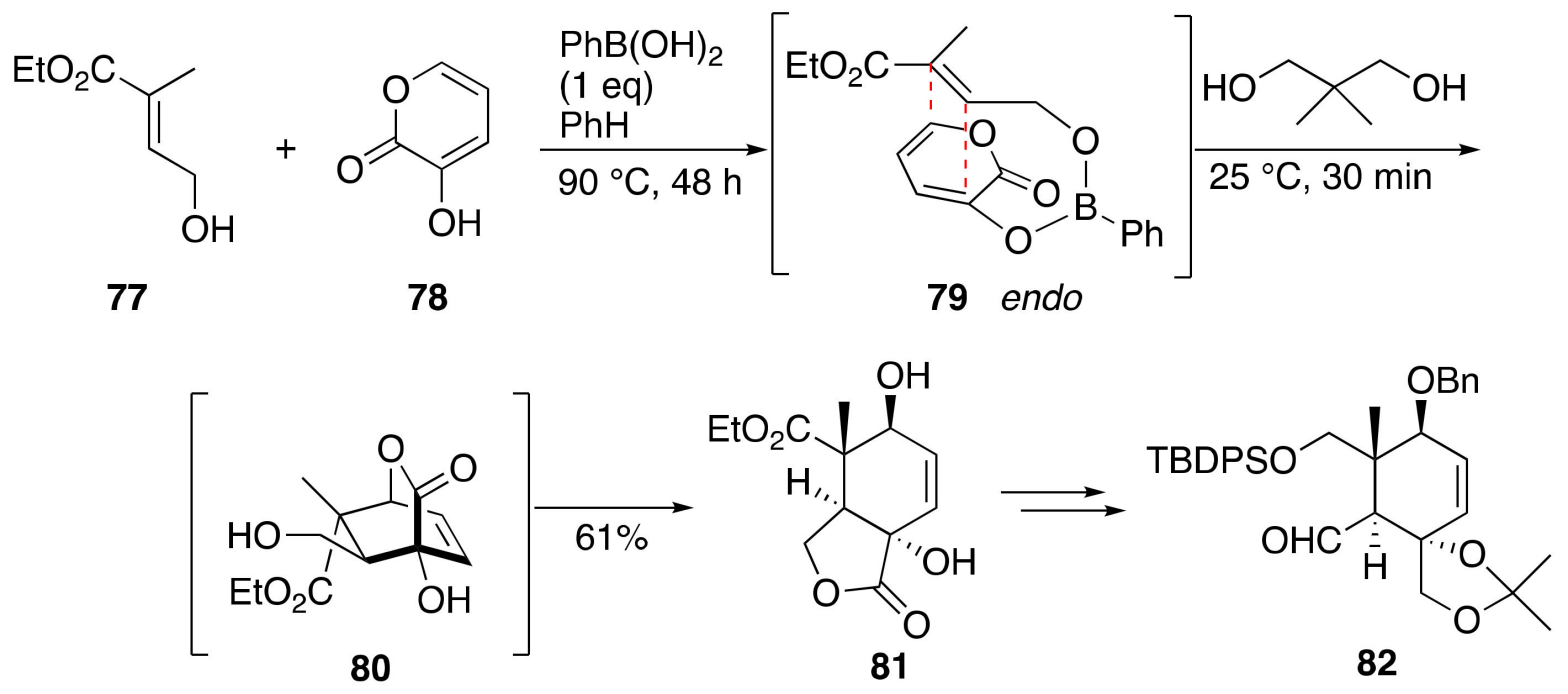
Nicolaou’s boron-templated Diels–Alder reaction in the total synthesis of taxol.

Nicolaou and co-workers reported a boron-templated Diels–Alder reaction as part of their monumental total synthesis of taxol (**5**) (Scheme 14) [24,25,26]. Since the unassisted intermolecular reaction between α,β-unsaturated ester (**77**) and 3-hydroxy-2-pyrone (**78**) was not successful in terms of both regioselectivity and chemical yield, both substrates (**77**, **78**) were combined with phenylboronic acid under heating conditions to generate the putative mixed borate ester (**79**) by dehydration and thermal Diels–Alder reaction, followed by treatment with 2,2-dimethyl-1,3-propanediol to afford bicyclic 𝛾-lactone (**81**) in a good yield. Compound **81** was successfully converted to the CD ring moiety (**82**) of taxol. In this case, the boron atom serves as a template, bringing the diene and dienophilic components together to enable an IMDA reaction, thus allowing the regiochemical course of the cycloaddition to be rigorously controlled.

### 4.3. Magnesium-Templated Diels–Alder Reactions

**Scheme 15:**
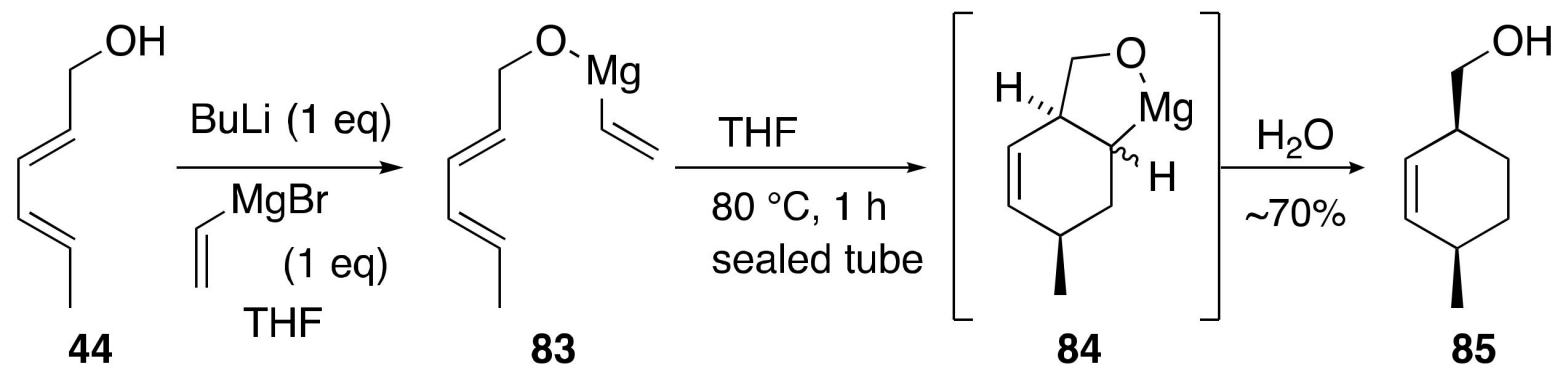
Stork’s temporary magnesium connection in a Diels–Alder reaction.

Magnesium can also be a temporary tether (Scheme 15) [63]. Stork and co-workers prepared a magnesium-templated intermediate from vinylmagnesium bromide and allylic alcohol (**44**) with butyl lithium. The mixture underwent a thermal Diels–Alder reaction to afford the cyclohexene derivative (**85**) a good yield. It is interesting to note that the thermal Diels–Alder reaction took place at a lower temperature than the silicon-templated reaction mentioned above. Cycloaddition occurred despite the fact that dienophile is formally a vinyl carbanion; indeed, it was highly facilitated.

### 4.4. Aluminum-Templated Diels–Alder Reactions

**Scheme 16:**
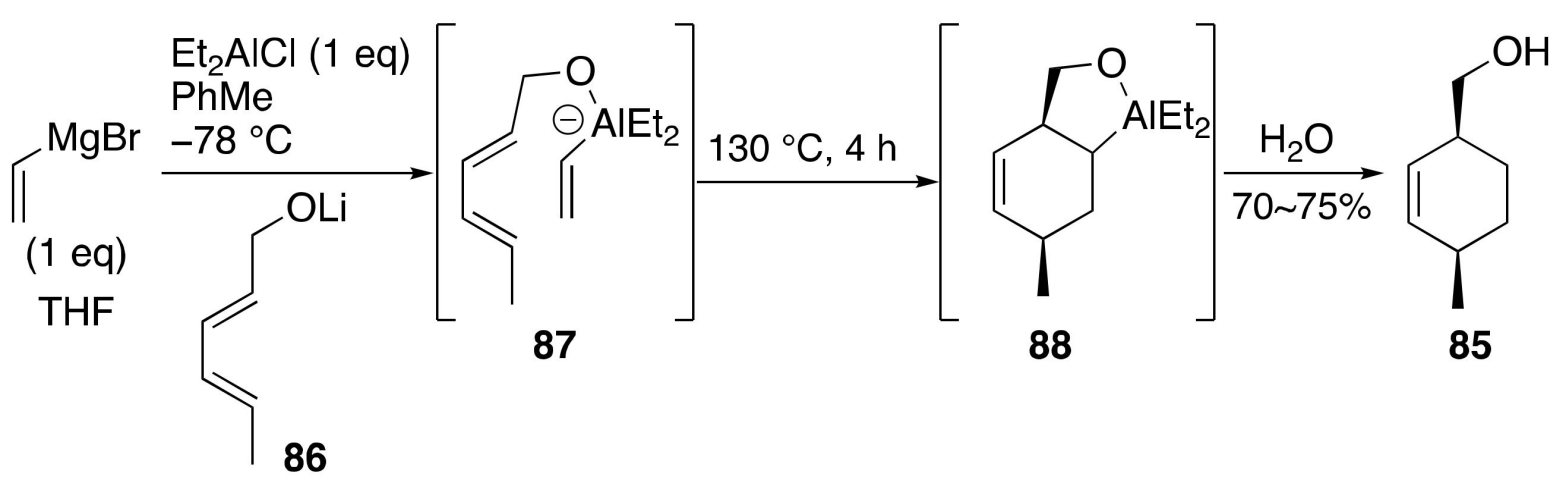
Stork’s aluminum-templated Diels–Alder reaction.

Stork’s group also reported an aluminum-templated Diels–Alder reaction in the above-mentioned paper on magnesium-templated Diels–Alder reactions (Scheme 16) [64]. The addition of vinylmagnesium bromide in THF to diethylaluminium chloride in toluene and the lithium alkoxide (**86**) followed by heating at 130 °C afforded the cycloadduct (**85**) a 70–75% yield. The concerted manner of the reaction was established through a comparison of the products, as in the case of the magnesium template.

**Scheme 17:**
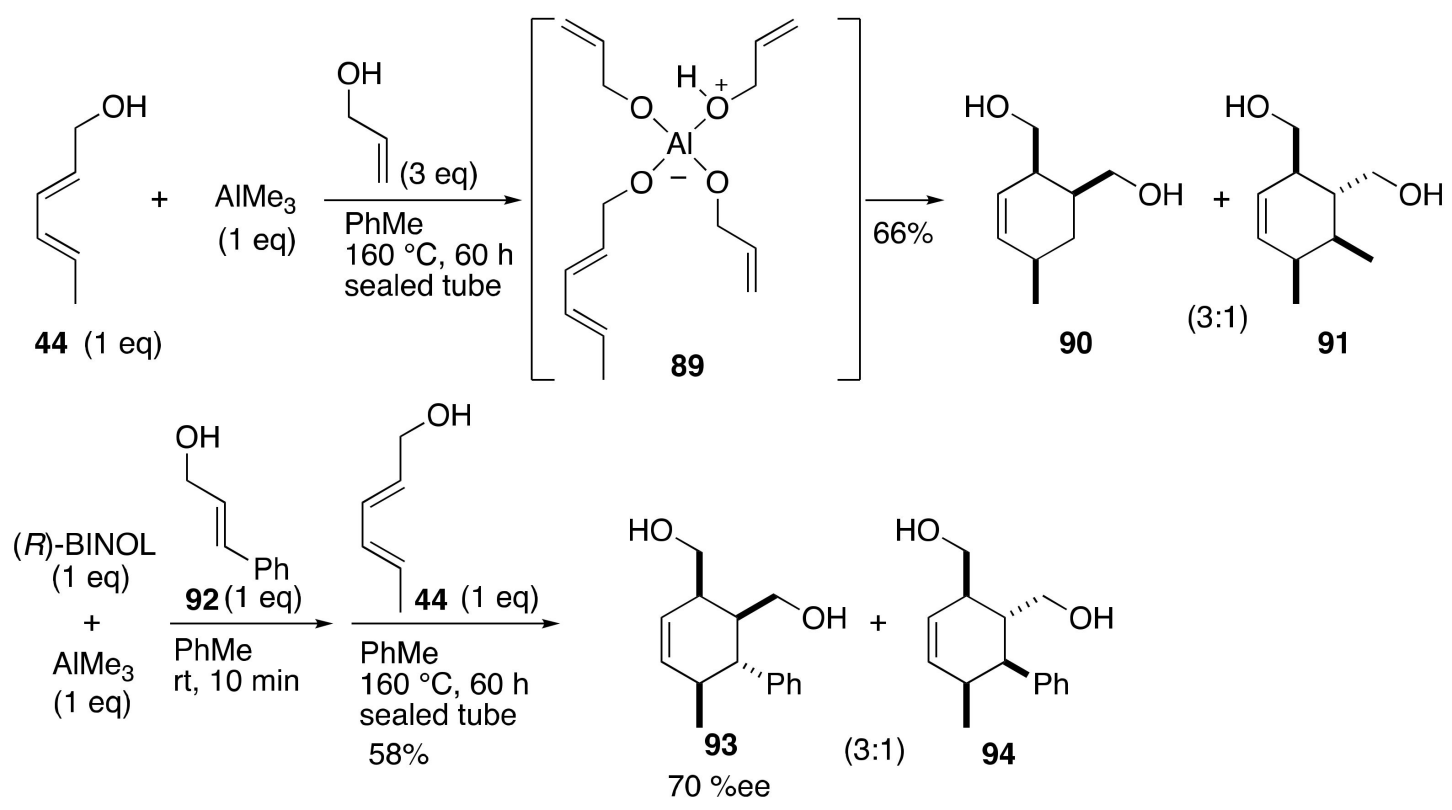
Olsson’s aluminum-templated Diels–Alder reaction.

Olsson and co-workers reported an aluminum-templated Diels–Alder reaction (Scheme 17) [64]. The aluminum-templated cycloaddition reaction of sorbic alcohol (**44**) and allyl alcohol at 160 °C for 60 h afforded a 3:1 diastereomeric mixture of cyclohexenes (**90**, **91**) in a 66% overall yield after hydrolytic work-up. They also attempted the asymmetric Diels–Alder reaction of **92** and **44** using (*R*)-BINOL to give a 3:1 mixture of compounds **93** and **94** as an optically active mixture. Notably, the major product had an *ee* of 70%.

### 4.5. Zinc-Templated Diels–Alder Reactions

**Scheme 18:**
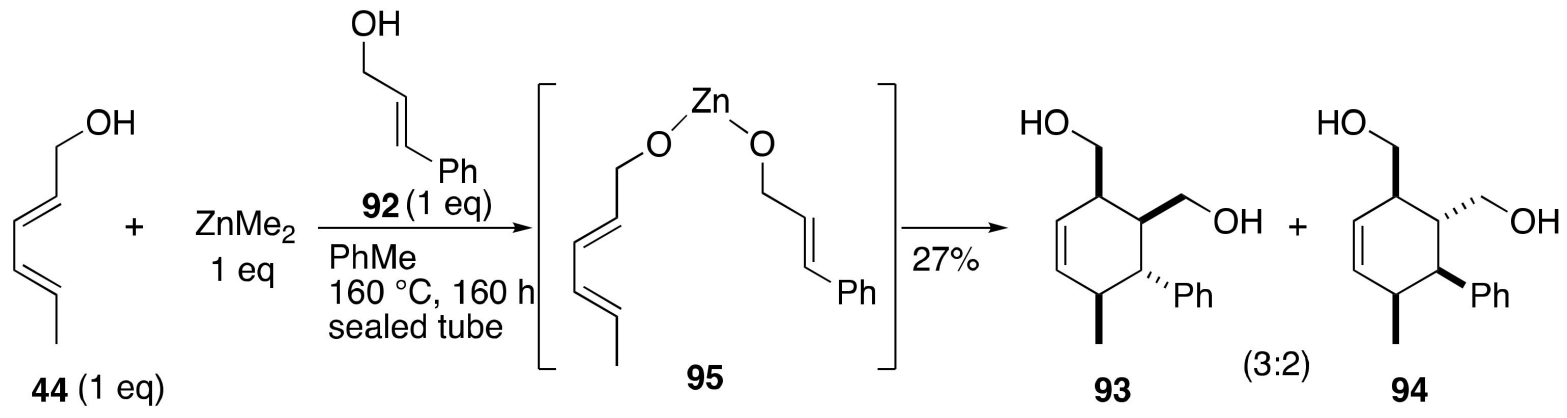
Olsson’s zinc-templated Diels–Alder reaction.

Olsson and co-workers also reported using zinc as a linker atom in their studies on templated Diels–Alder reactions (Scheme 18) [64]. In the zinc-templated reaction of sorbic alcohol (**44**) and allylic alcohol **92** at 160 °C for 160 h, the same complete regioselectivity was observed as with aluminum. However, both the yield and diastereoselectivity were lower than those for the aluminum-mediated reaction.

## 5. Lewis-Acid-Templated Diels–Alder Reactions

The aforementioned templated Diels–Alder reactions involving the linkage of a diene and a dienophile perfectly control the regiochemistry of the products, but they require heating, and additional transformations may be necessary to remove the tethered atom. However, if a Lewis acid is used as a template, the reaction can be carried out at room temperature or lower, and the work-up is simplified.

### 5.1. Aluminum Lewis-Acid-Templated Diels–Alder Reactions

**Scheme 19:**
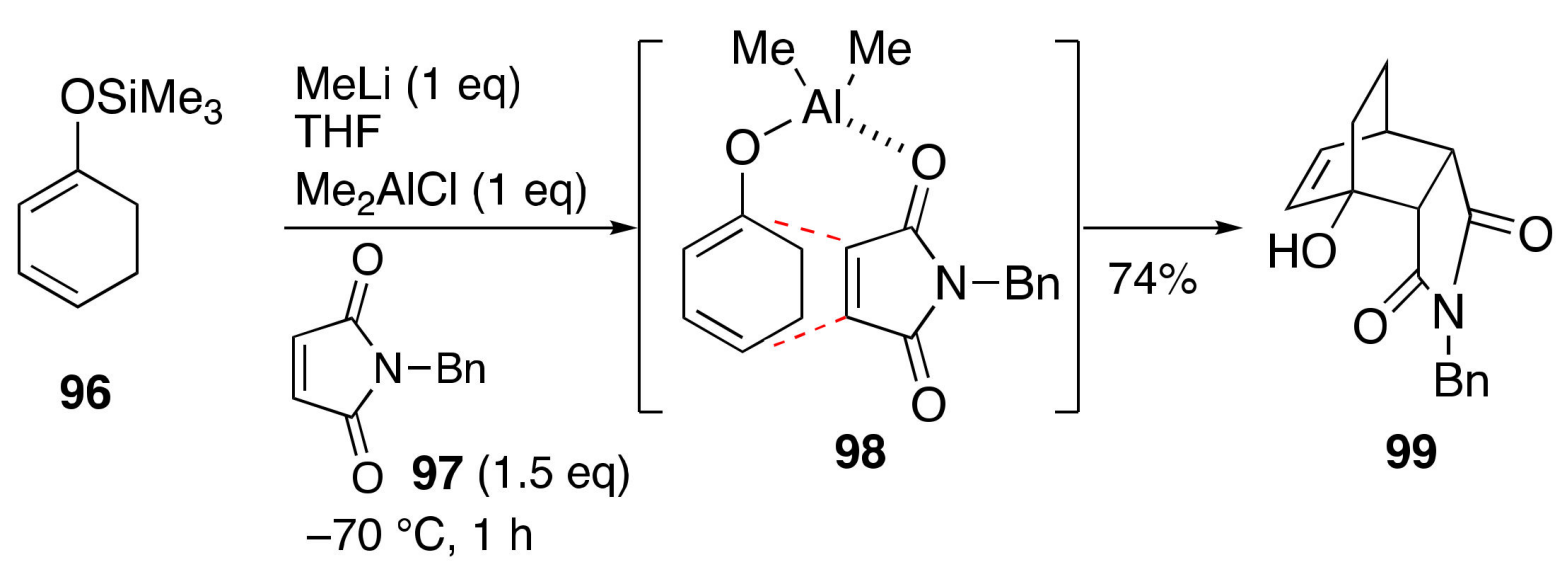
Bienaye’s Lewis-acid-templated Diels–Alder reaction.

Bienayé and co-workers reported the Diels–Alder reaction of suitable dienes covalently bonded to a Lewis acid, such as dimethylaluminum dienolates, allowing rapid and selective Diels–Alder cycloadditions with various dienophiles (Scheme 19) [65]. In the case of *N*-benzylmaleimide (**97**), the cyclohexadienylsilyl ether (**96**) was lithiated, followed by the addition of dimethylaluminum chloride to form an alkoxyaluminum complex. A further addition of maleimide brought the substrates closer together (**98**). The Diels–Alder reaction was thus efficient even at low temperatures, yielding the corresponding tricyclic product **99** in a 74% yield. In these processes, cooperation between enthalpic factors, such as the activation of the dienophile, and entropic factors, such as the pre-association of the reactants, is responsible for the high reactivities and regioselectivities observed.

### 5.2. Titanium Lewis-Acid-Mediated Diels–Alder Reactions

**Scheme 20:**
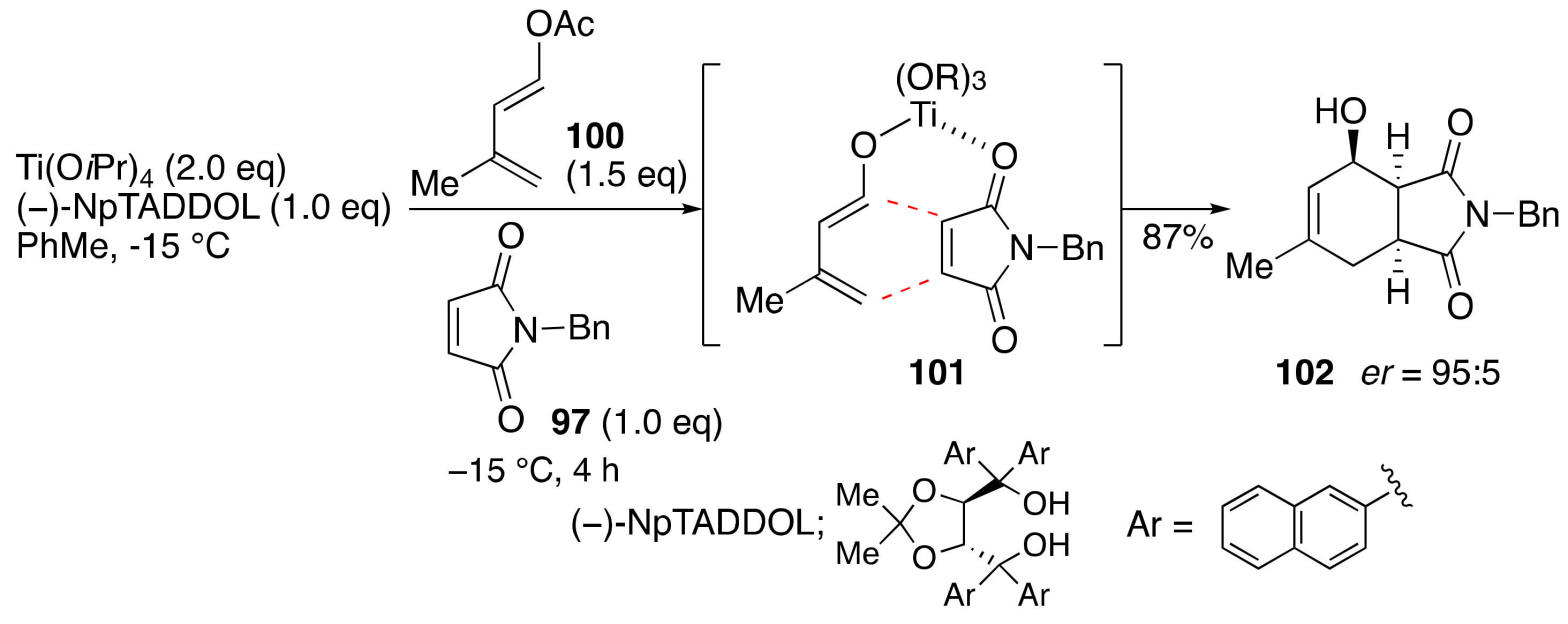
Bienaye’s Lewis-acid-templated Diels–Alder reaction.

Bienayé also reported an enantioselective Diels–Alder cycloaddition using a titanium Lewis acid template (Scheme 20) [66]. In this particular case, a mixture of Ti(O*i*Pr)_4_ and chiral NpTADDOL was treated with 1-acetoxyisoprene (**100**) and *N*-benzylmaleimide (**97**) at −15 °C for 4 h to afford the cycloadduct **102** a good yield and an enantiomeric ratio of 95:5. Although a stoichiometric amount of chiral Lewis acid template must be used, this method allows the reaction of difficult dienophiles such as maleimides with high selectivity under mild conditions.

### 5.3. Magnesium Lewis-Acid-Templated Diels–Alder Reactions

**Scheme 21:**
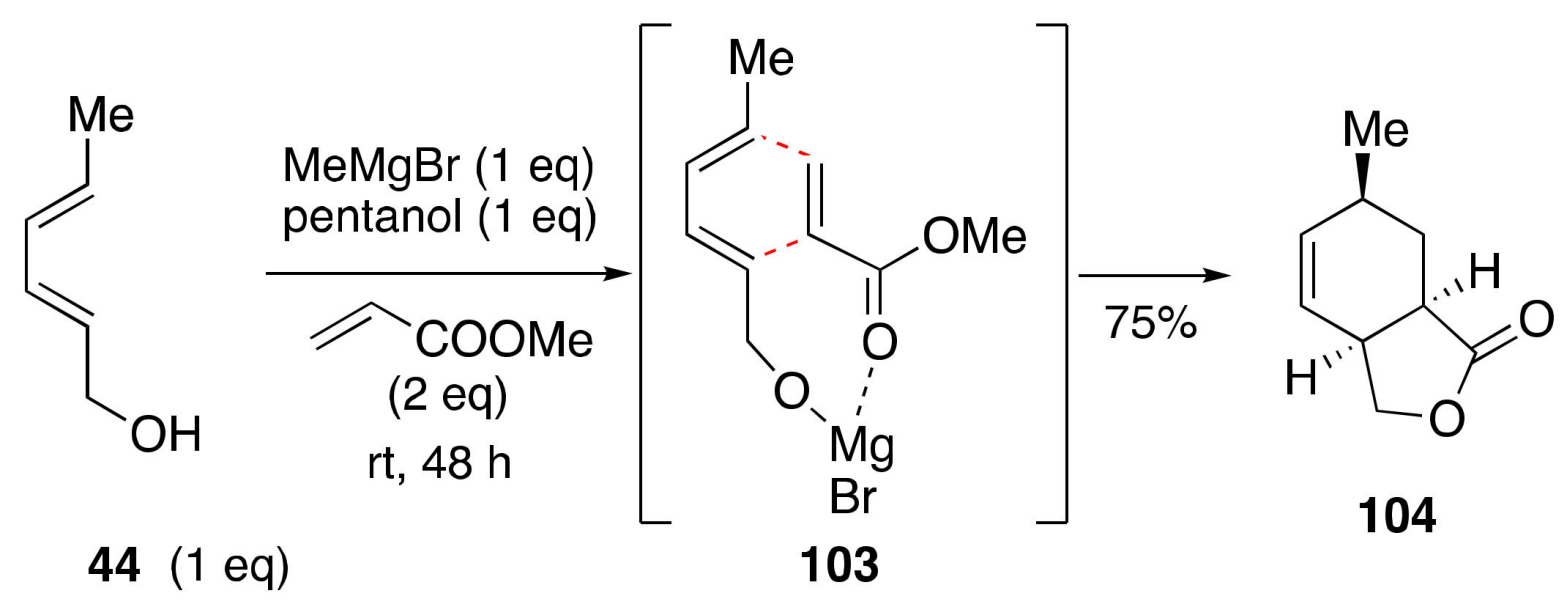
Ward’s Lewis-acid-templated Diels–Alder reaction.

Ward and co-workers demonstrated a new strategy to control Diels–Alder reactions based on a Lewis-acid-catalyzed reaction of a self-assembled complex, termed an LACASA-DA reaction, using magnesium (Scheme 21) [67]. The magnesium salt caused bimolecular proximity, leading to the Diels–Alder reaction and spontaneous lactonization to produce bicyclic lactone **104** in a high yield with excellent regio- and diastereoselectivity. The dienophile was activated by magnesium alkoxide, so the reaction proceeded without heating.

**Scheme 22:**
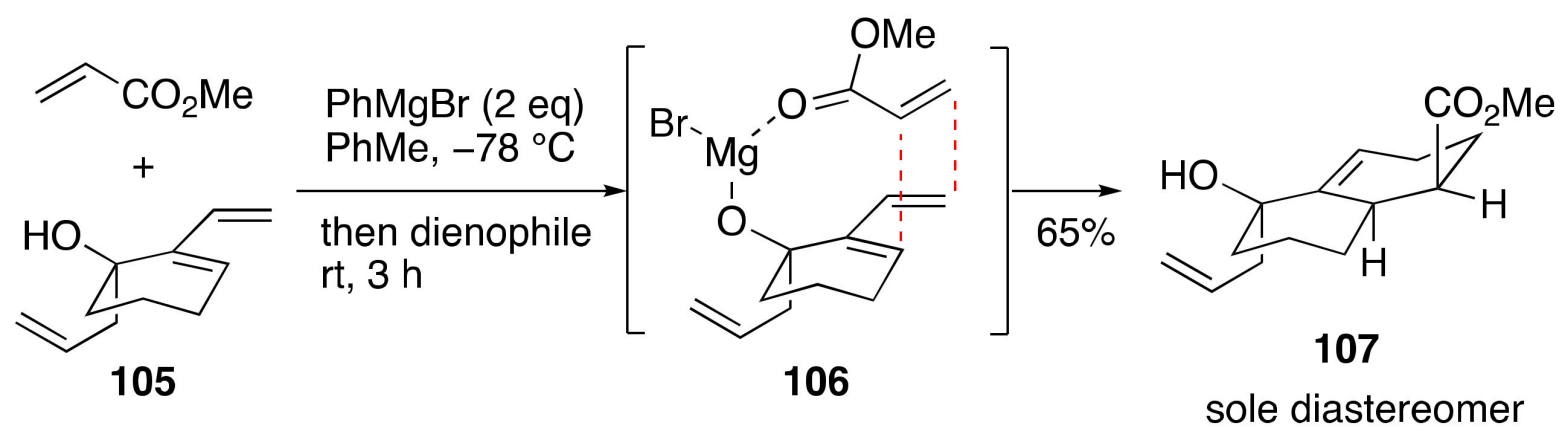
Barriault’s Lewis-acid-templated Diels–Alder reaction.

Barriault and co-workers performed a Lewis-acid-templated Diels–Alder reaction using phenylmagnesium bromide (Scheme 22) [68,69,70]. In this reaction, the hydroxyl group in diene **105** underwent a reaction with the Grignard reagent to form magnesium alkoxide (**106**) in situ. Methyl acrylate was then added and the mixture was stirred at room temperature for 3 h, yielding the cycloadduct (**107**) in 65% as a sole diastereomer.

**Scheme 23:**
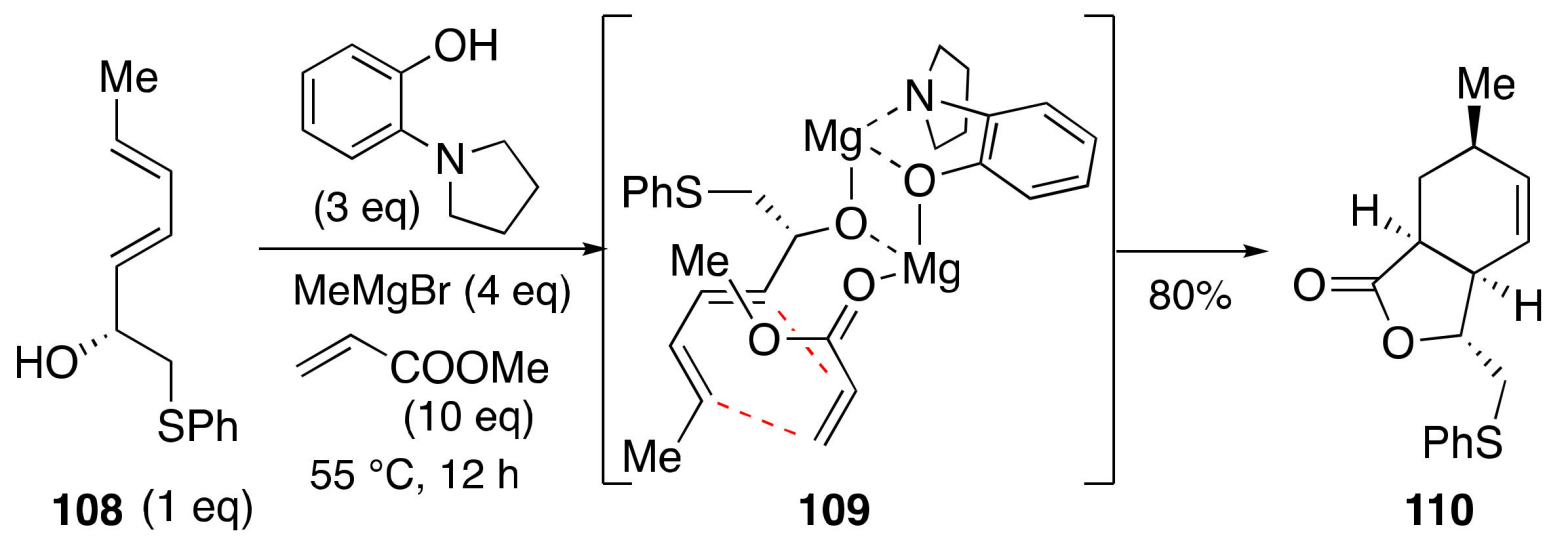
Nicolaou’s Lewis-acid-templated Diels–Alder reaction.

Nicolaou’s group modified Ward’s method using a template prepared from a 2-aminophenol derivative and a Grignard reagent (Scheme 23) [24,25,26]. In this case, a bicyclic lactone (**110**) was constructed stereoselectively through coordination with a secondary hydroxyl group of substrate **108**. They achieved the total synthesis of abyssomicin C using the bicyclic lactone as a key intermediate. A similar reaction has also been reported by Georgiadis et al. [71].

### 5.4. Bimetallic Lewis-Acid-Templated Diels–Alder Reactions

**Scheme 24:**
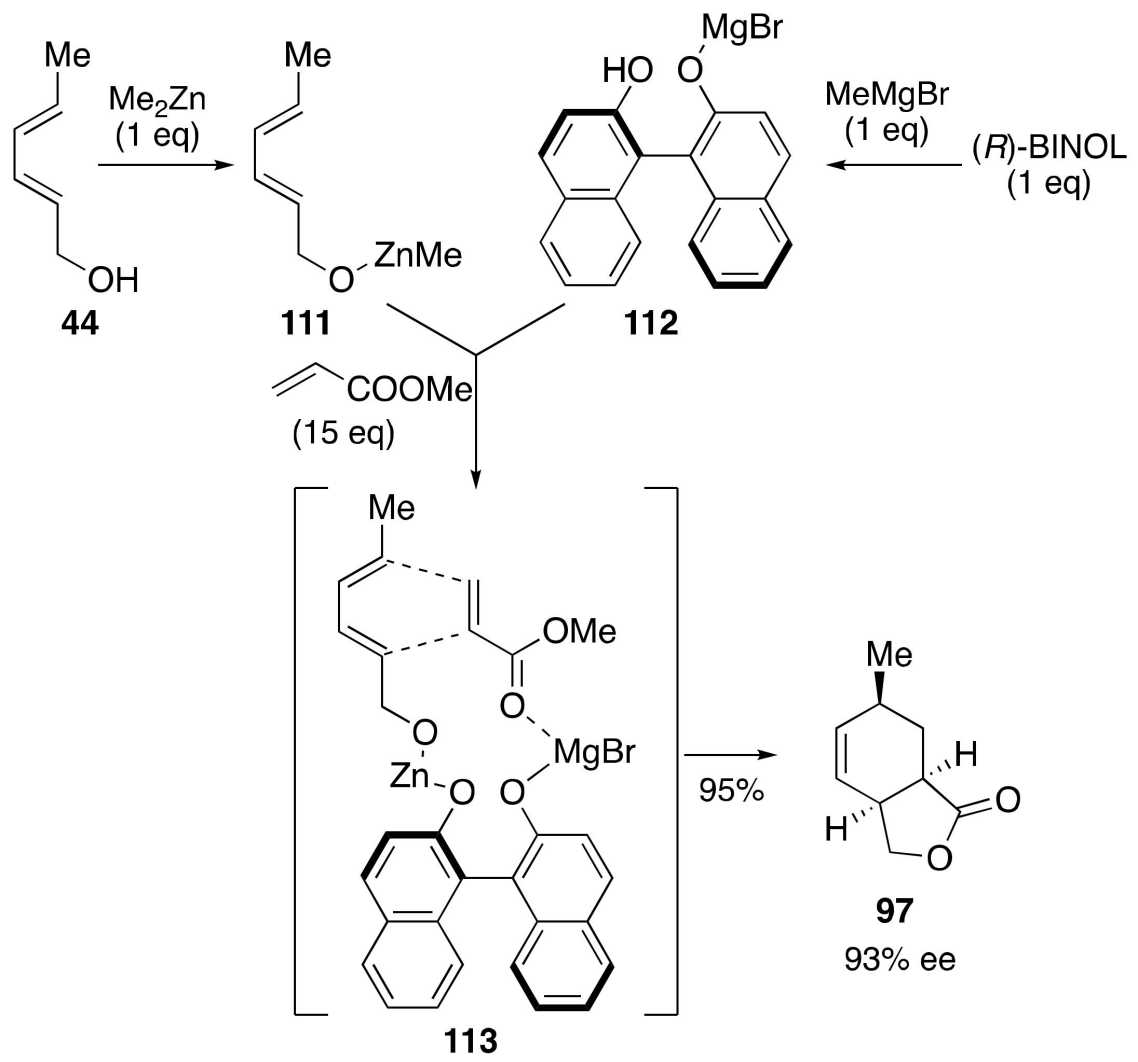
Ward’s enantioselective Diels–Alder reaction.

Ward and co-workers reported an enantioselective LACASA-DA reaction based on a bimetallic Lewis acid (Scheme 24) [72]. In this particular case, 2,4-pentadienol (**44**) was treated with an equimolar amount of ZnMe_2_ and optically active BINOL was treated with an equimolar amount of MeMgBr; then, the mixtures were combined and methyl acrylate was added. Under these conditions, the Diels–Alder reaction proceeded smoothly and subsequent lactonization produced bicyclic lactone **97** in an excellent yield with high regioselectivity, stereoselectivity, and enantioselectivity. It was also found that the reaction also proceeded smoothly with catalytic amounts of Lewis acid, but the turnover was very low.

Notably, all of these Lewis-acid-templated reactions required stoichiometric amounts of reagents to achieve acceptable conversions, while similar reactions with low catalyst loadings were yet to be achieved.

## 6. Catalytic Chiral-Lewis-Acid-Templated Diels–Alder Reactions

### 6.1. Lewis-Acid-Templated Diels–Alder Reactions Using H_8_-BINOL

**Scheme 25:**
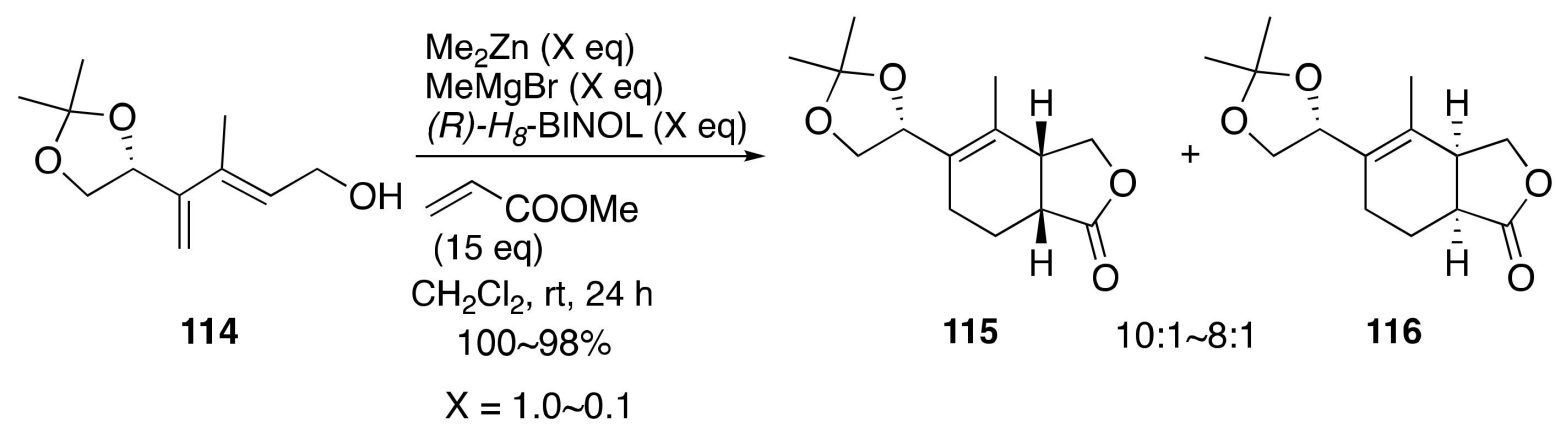
Lewis-acid-templated Diels–Alder reaction of **114** and methyl acrylate using *H_8_*-BINOL.

Ishihara and co-workers have developed the method of Ward into a catalytic format (Scheme 25) [73]. First, they studied the Diels–Alder reaction of **114** and 15 eq. of methyl acrylate using 1 eq. of (*R*)-BINOL as the chiral ligand according to Ward’s protocol. The reaction proceeded at room temperature for 3.5 h to yield a 4.8:1 diastereoisomeric mixture of bicyclic lactones **115** and **116**. Conversely, the reaction with (*R*)-5,5′,6,6′,7,7′,8,8′-octahydro-1,1′-bi-2-naphthol (*H_8_*-BINOL) in place of (*R*)-BINOL exhibited higher selectivity, giving **115** and **116** in a 98% combined yield and a ratio of 10:1. Surprisingly, the reaction mediated by 0.5 eq. of the bimetallic complex with *H_8_*-BINOL was completed within 2 h to give **115** and **116** in a 99% yield and with a diastereoselectivity that was similarly high to that observed for the stoichiometric reaction. After investigation of the reaction under lower catalyst loading, it was found that the use of even 0.1 eq. of the catalyst resulted in high selectivity.

### 6.2. Catalytic Lewis-Acid-Templated Diels–Alder Reactions

**Scheme 26:**
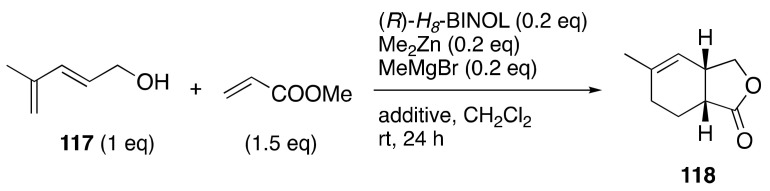
Lewis-acid-templated Diels–Alder reaction of **117** and methyl acrylate using *H_8_*-BINOL.

The cycloaddition reaction of 4-methyl-2,4-pentadienol (**117**), an achiral substrate, was also investigated (Scheme 26). When **117** was reacted with methyl acrylate using 0.2 eq. of the bimetallic complex with *H_8_*-BINOL at room temperature for 3 d, the cycloaddition proceeded with moderate enantioselectivity to afford bicyclic γ-lactone **118** in an 83% yield and 52% *ee*. However, the addition of 4Å molecular sieves and decreasing the amount of methyl acrylate markedly improved the enantioselectivity. Consequently, the chiral template reaction of **117** and 1.5 eq. of methyl acrylate using *H_8_*-BINOL in the presence of 4Å molecular sieves provided **118** in a 98% yield and 92% *ee*.

### 6.3. Scope and Limitation of the Catalytic Lewis-Acid-Templated Diels–Alder Reaction

**Scheme 27:**
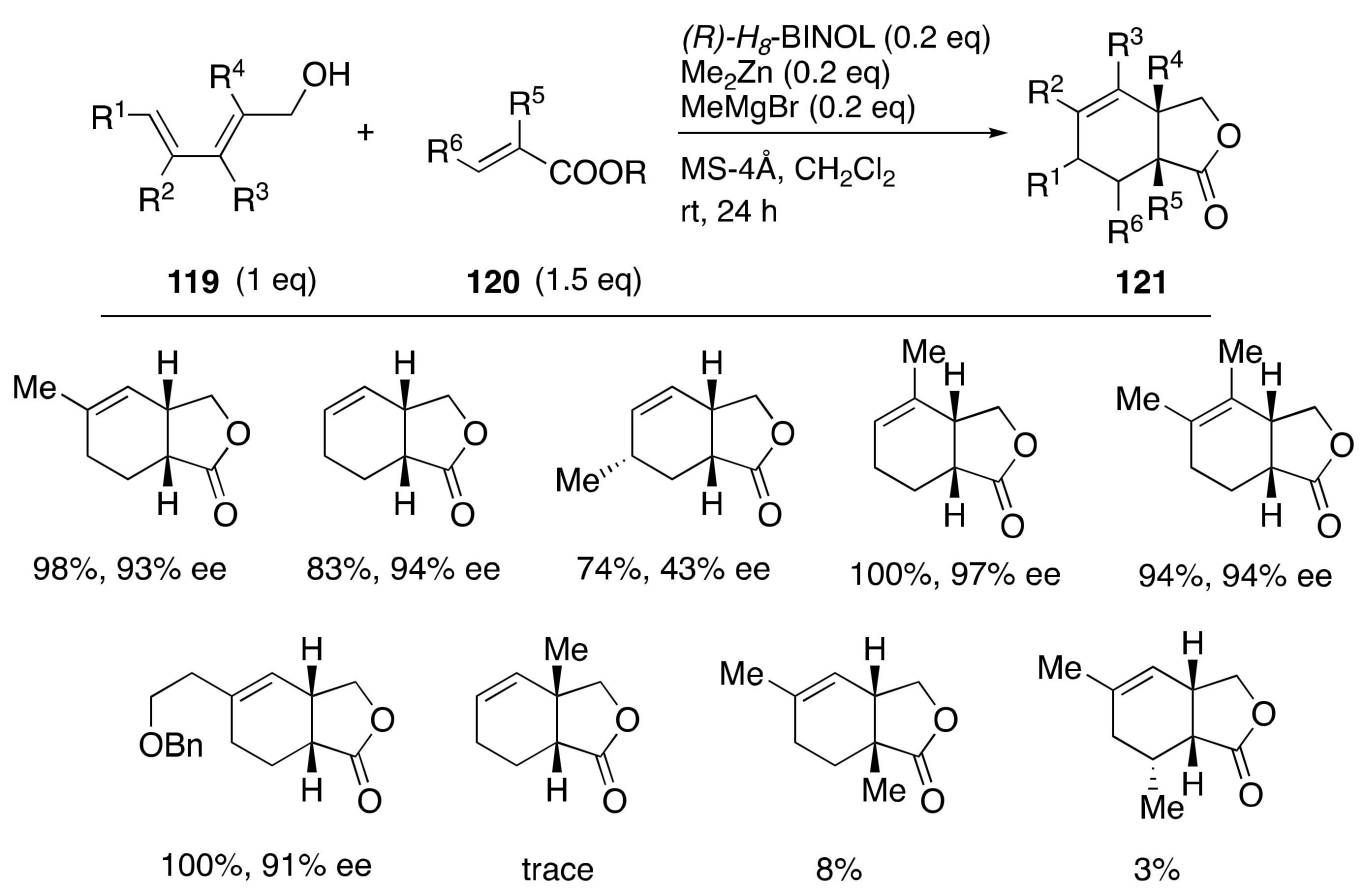
Ishihara’s catalytic Lewis-acid-templated Diels–Alder reaction.

The generality of this protocol was then investigated (Scheme 27). It was found that the Lewis-acid-template-catalyzed reaction is applicable to various 2,4-pentadienol substituents, and both the yields and *ee* values are high, especially for substituents (R^3^ and R^2^) at the 3 and 4 positions. Conversely, the reaction of 2,4-hexadienol showed poor enantioselectivity, although the yield was acceptable. Furthermore, 2-methyl-2,4-pentadienol was found to be a less reactive substrate, possibly due to steric factors.

**Scheme 28:**
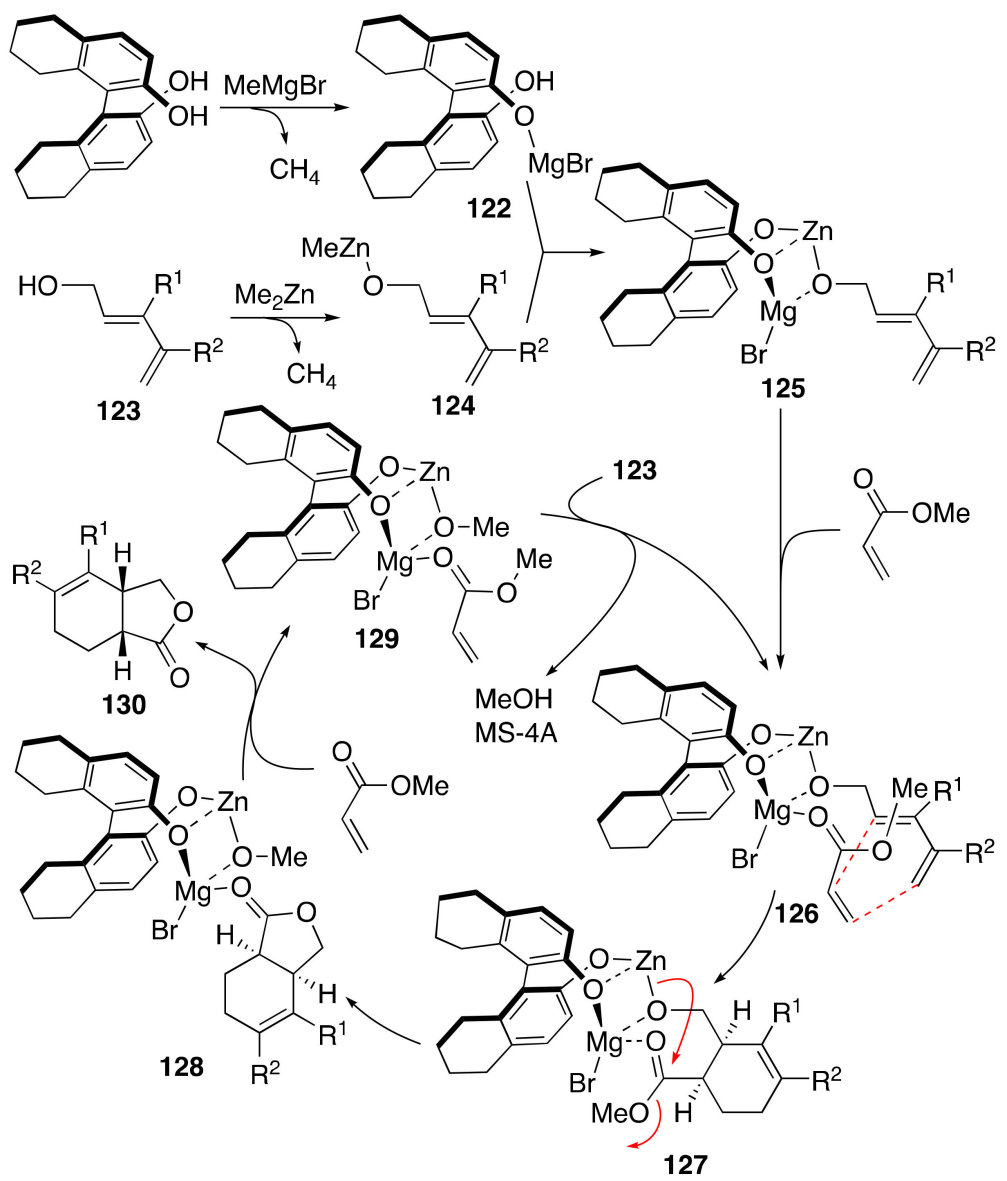
Plausible mechanism of the Lewis-acid-catalyzed Diels–Alder reaction.

A plausible mechanism for the catalytic Diels–Alder reaction is shown in Scheme 28. In the catalytic cycle of the reaction, the coordination of magnesium octahydrobinapthoate **122** with zinc dienolate **124** initially forms the bimetallic Lewis acid **125**, which reacts with methyl acrylate to give intermediate **126**. The tethered intermediate **126** undergoes a Diels–Alder reaction in *endo* mode (**127**), followed by lactonization (**128**). The ligand exchange of **128** with methyl acrylate yields γ-lactone product **130**. The second exchange of the resulting **129** with dienol **123** regenerates **126** and releases methanol. 4Å or 5Å molecular sieves are required for the catalytic reaction, possibly because they capture the methanol produced during lactonization to accelerate the catalytic cycle. The reason for the higher catalytic efficiency of *H_8_*-BINOL than BINOL is not clear, but the larger dihedral angle of *H_8_*-BINOL may play an important role in smooth ligand exchange in the catalytic cycle.

## 7. Application of Lewis-Acid-Templated Diels–Alder Reactions to Natural Product Synthesis

### 7.1. Nicolaou’s Total Synthesis of Abyssomicin C

**Scheme 29:**
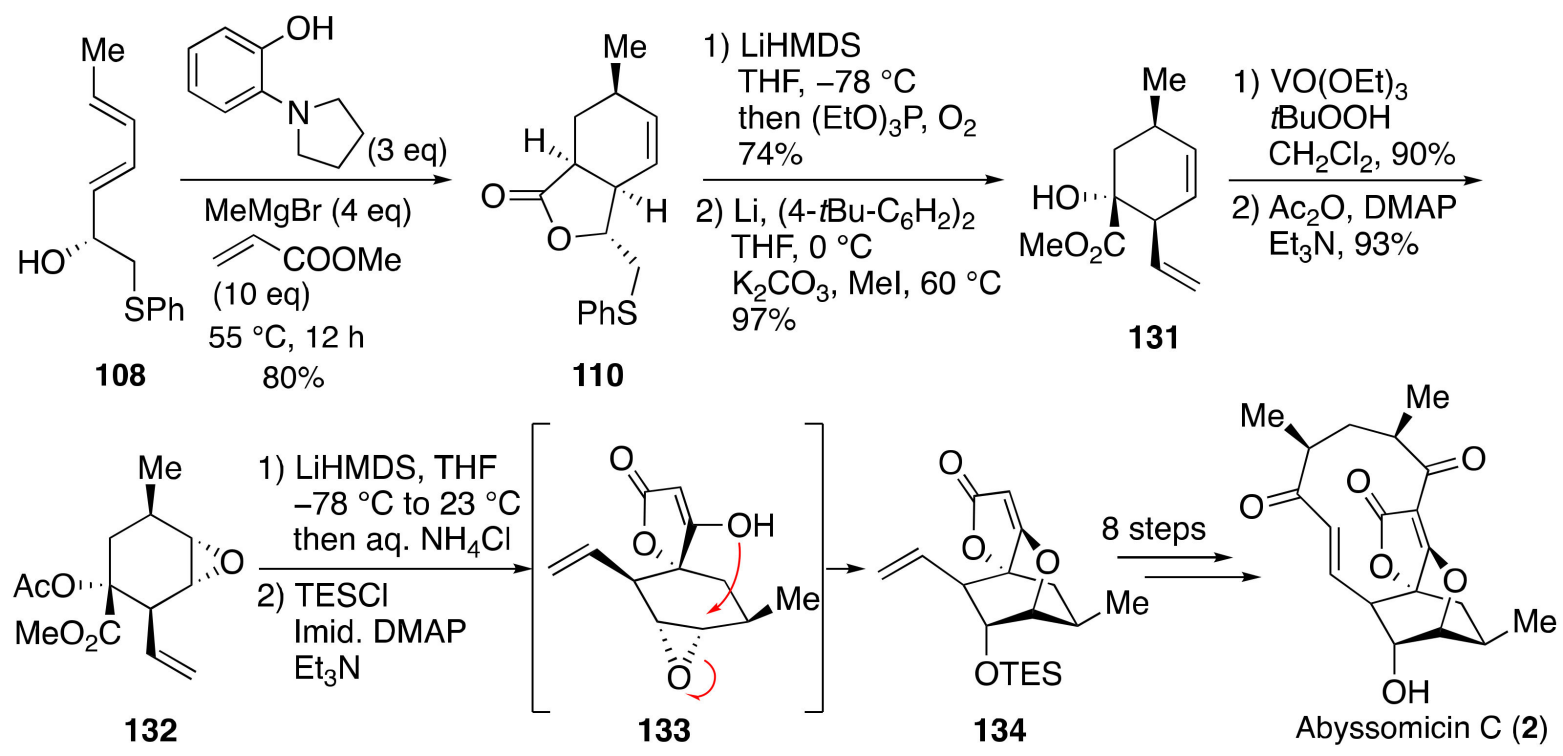
Nicolaou’s total synthesis of abyssomicin C.

Abyssomicin C (**2**) was isolated from the actinomycete *Verrucosispora* strain AB 18-032. This natural product exhibits strong activity against both methicillin-resistant *Staphylococcus aureus* (MRSA; 4 μg mL^−1^) and vancomycin-resistant *Staphilococcus aureus* (VRSA; 13 μg mL^−1^) [74,75,76]. One of the synthetic strategies developed by Nicolaou and co-workers is the early-stage formation of the core of abyssomicin C utilizing an optically active bicyclic lactone (Scheme 29) [24,25,26]. Thus, the bicyclic lactone **110** was prepared diastereoselectively from chiral alcohol **108** via their unique Lewis-acid-templated Diels–Alder reaction. The resulting bicyclic product was deprotonated with a base and quenched with O_2_ and (EtO)_3_P to give α-hydroxy lactone. The treatment of the phenylthiomethyl γ-lactone with a radical anion species led to the ring opening of the lactone with concomitant olefin formation, followed by methyl esterification to give hydroxy diene **131**. Compound **131** was then subjected to vanadium-directed epoxidation and subsequent acetylation to afford epoxide **132**. Dieckmann condensation and regioselective ring opening of **132** then afforded tetracyclic intermediate **134**. Compound **134** was converted to the target molecule in eight steps.

### 7.2. Roush’s Stereoselective Synthesis of the Hirsutellone Core

**Scheme 30:**
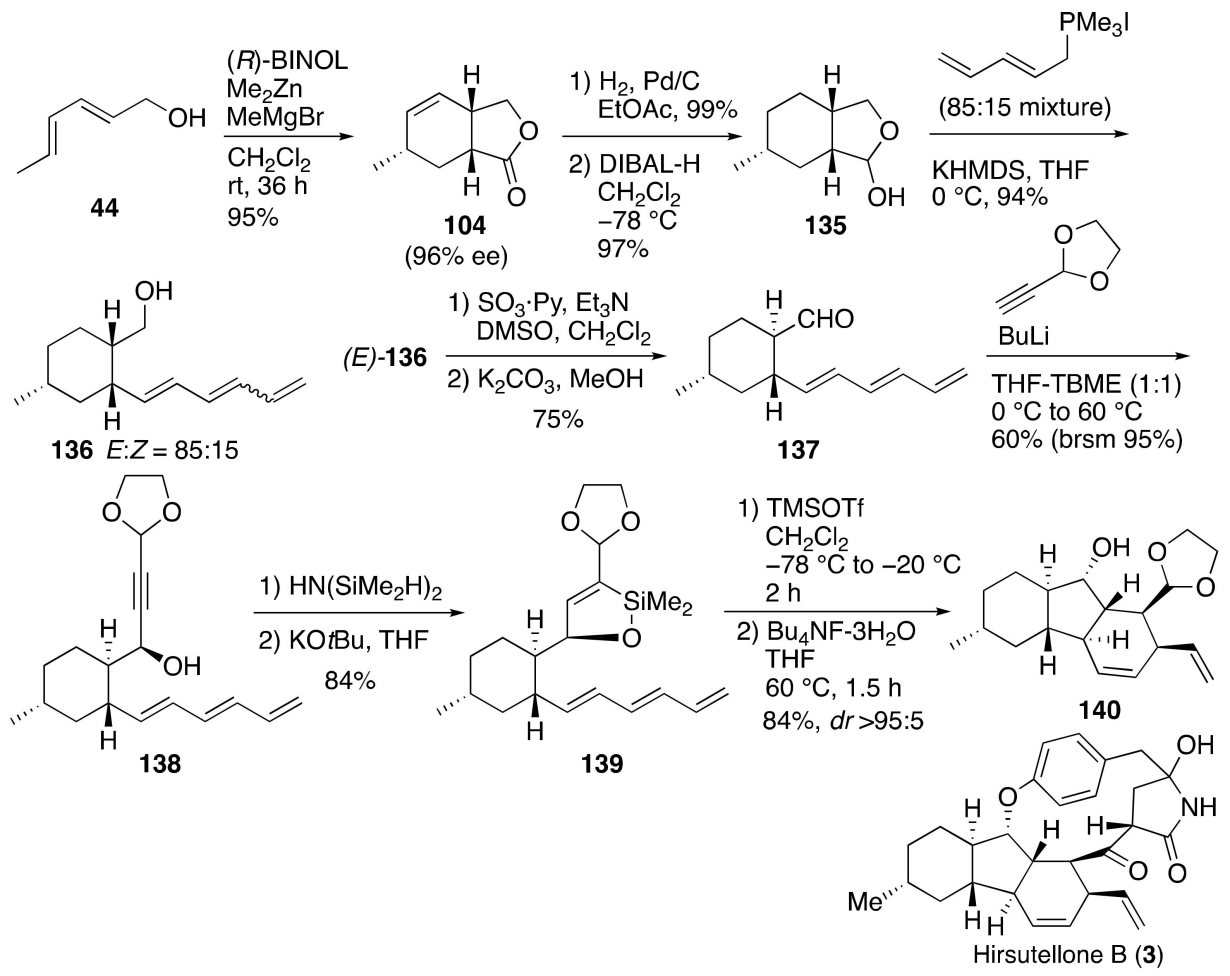
Roush’s synthesis of the hirsutellone core.

The hirsutellones are a family of structurally interesting polyketides with a decahydrofluorene core which were isolated from the insect pathogenic fungus *Hirsutella nivea* BCC 2594 [77]. These natural products exhibit potent antitubercular activity. Roush and co-workers have reported the stereoselective synthesis of the molecule’s core (Scheme 30) [28]. The synthesis of the common hirsutellone core started with Ward’s enantioselective Lewis-acid-templated Diels–Alder reaction. The resulting chiral bicyclic lactone (**104**) was readily converted by hydrogenation and DIBAL-H reduction to lactol **135**, which was subjected to a Wittig reaction to give the cyclohexane derivative **136**. The pure *E*-product was then oxidized to the aldehyde and treated with K_2_CO_3_, which resulted in a smooth epimerization of the aldehyde to exclusively give the desired aldehyde (**137**). Following alkynation, propargyl alcohol **138** was subjected to hydrosilylation, yielding siloxacyclopentene **139**. The product underwent a Lewis-acid-mediated IMDA reaction to afford the hirsutellone core.

### 7.3. Jin’s Synthesis of Truncated Superstolide A

**Scheme 31:**
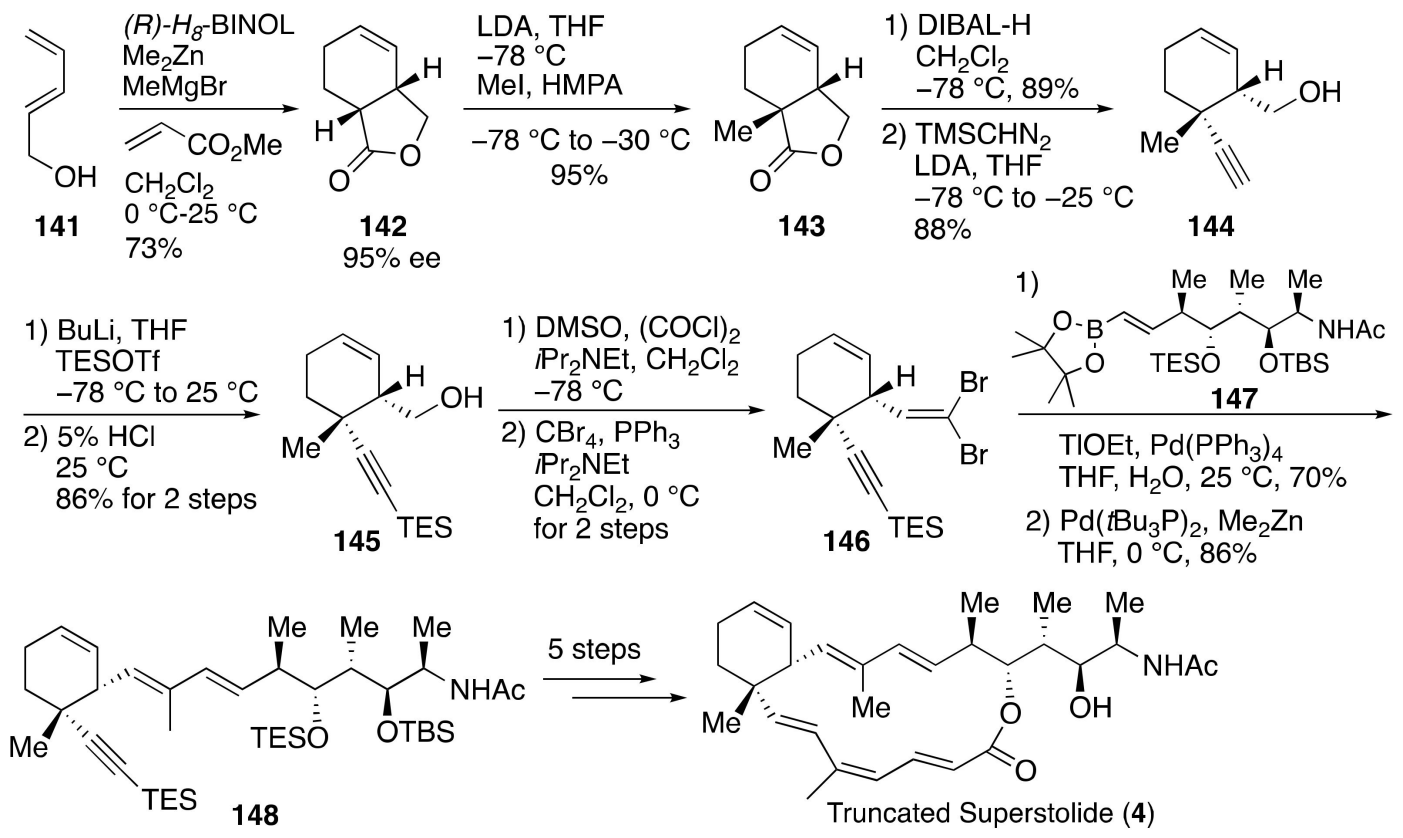
Jin’s synthesis of truncated superstolide.

Superstolides A and B were isolated in minute amounts from the deep-sea sponge *Neosiphonia superstes*. Both superstolides A and B show potent antiproliferative effects against several tumor cell lines [78,79]. Jin and co-workers have reported the design and synthesis of a truncated superstolide A, in which the *cis*-fused functionalized decalin is simplified to a cyclohexene ring, while the 16-membereed macrolactone remains intact (Scheme 31) [29]. To prepare the cyclohexene, they selected Ward’s chiral-Lewis-acid-templated Diels–Alder cycloaddition as the first step. The resulting optically active lactone (**142**) was subjected to α-methylation and DIBAL-H reduction followed by Sayfeth–Gilbert alkynation to afford a functionalized cyclohexene **144**. After TES protection, alcohol **145** was oxidized to the corresponding aldehyde **146**, which was then converted to the geminal dibromo compound **146**. Suzuki coupling between compound **146** and vinyl boronate **147** stereoselectivity provided the vinyl bromide. The subsequent Negishi coupling between the resulting vinyl bromide and Me_2_Zn gave the desired trisubstituted olefin **148** in an 86% yield. This intermediate **148** was successfully converted to the target molecule in five steps.

### 7.4. Ishihara’s Synthesis of the Upper Segment of Spirolides A and B

**Scheme 32:**
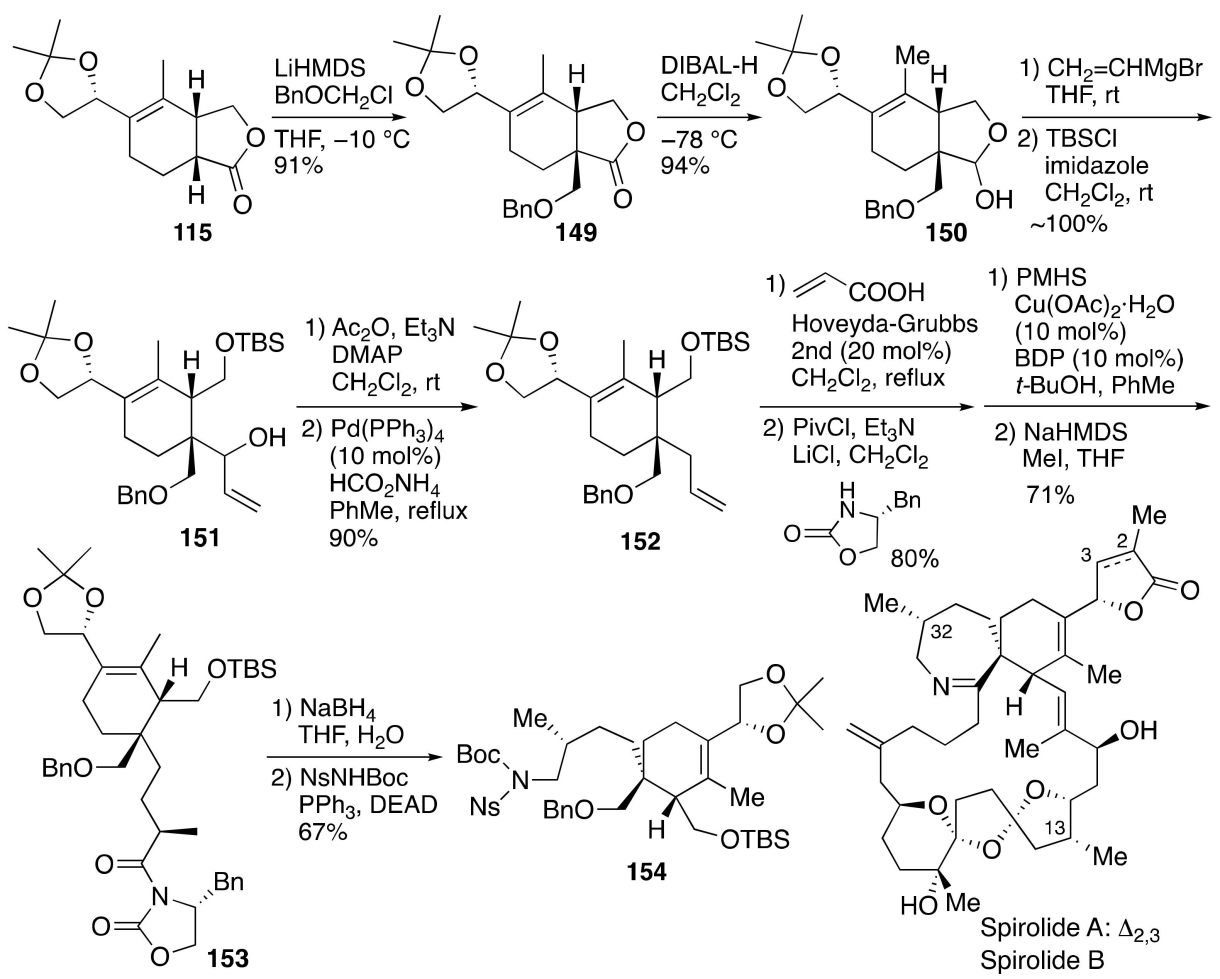
Ishihara’s synthesis of the upper fragment of spirolides A and B.

Since 1995, a number of marine natural products containing the spiroimine moiety have been found to cause food poisoning. Spirolides [80,81] are marine toxins derived from bivalve mollusks and have similar structures to natural products such as pinnatoxin [82] and gymnodimine [83] (Figure 2). In particular, 13-desmethylspyrrolide C is one of the most potent neurotoxins among non-peptide natural products [84,85]. These natural products exhibit antagonistic activity against the nicotinic acetylcholine receptor (nAChR). Ishihara and co-workers reported the synthesis of the spirocyclic core of spirolides A and B starting from compound **115**, which was accessed by a Lewis-acid-template-catalyzed Diels–Alder reaction (Scheme 32) [38]. A benzyloxymethyl group was stereoselectively introduced at the α-position of the lactone, which was then converted to lactol **150** through DIBAL-H reduction. Subsequently, the introduction of the vinyl group using a Grignard reagent followed by TBS protection of the primary hydroxyl group afforded **151**. The acetylation of the secondary hydroxyl group of **151** and subsequent palladium-catalyzed reductive deacetoxylation afforded allylic product **152** in good yields. Cross-metathesis was used to introduce the oxazolidinone, followed by hydrogenation by Lipshutz 1,4-reduction and stereoselective methylation by Evans alkylation to give compound **153**. The removal of the asymmetric auxiliary group and Fukuyama amine synthesis led to the synthesis of the upper segment of spirolide A and B (**154**).

### 7.5. Landais’s Synthesis of the Spirocyclic Core of Demethylspirolide C and Gymnodimine

**Scheme 33:**
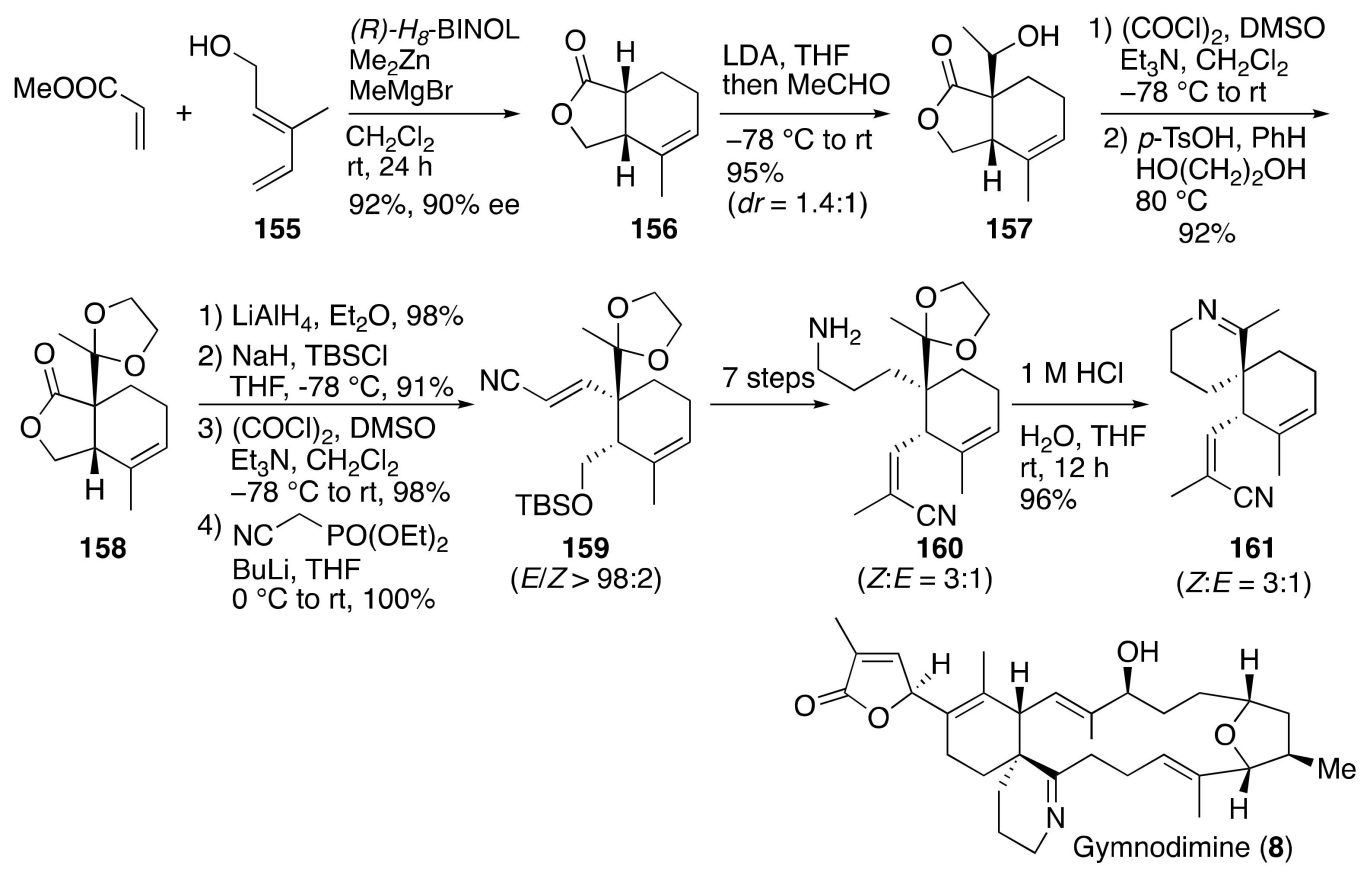
Landais’s synthesis of the spirocyclic core of demethylspirolide C and gymnodimine.

Landais and co-workers also reported an approach to natural products containing spiroimine, desmethylspirolide C, [81] and gymnodimine [83] (Scheme 33) [39]. They prepared the bicyclic 𝛾-lactone **156** in a good yield with high enantioselectivity via Ishihara’s Lewis-acid-template-catalyzed Diels–Alder reaction. The bicyclic product **156** was α-alkylated by an aldol reaction followed by oxidation and ketalization to afford **158**. Lactone **158** was subjected to LiAlH_4_ reduction to give the diol, which was protected by a TBS group followed by oxidation and Horner–Wadsworth–Emmons reactions to afford compound **159** in a good yield. Compound **159** was converted in seven steps to compound **160**, which was exposed to HCl to afford the spirocyclic imine product **161** an excellent yield.

### 7.6. Gao’s Total Synthesis of Farnesin

**Scheme 34:**
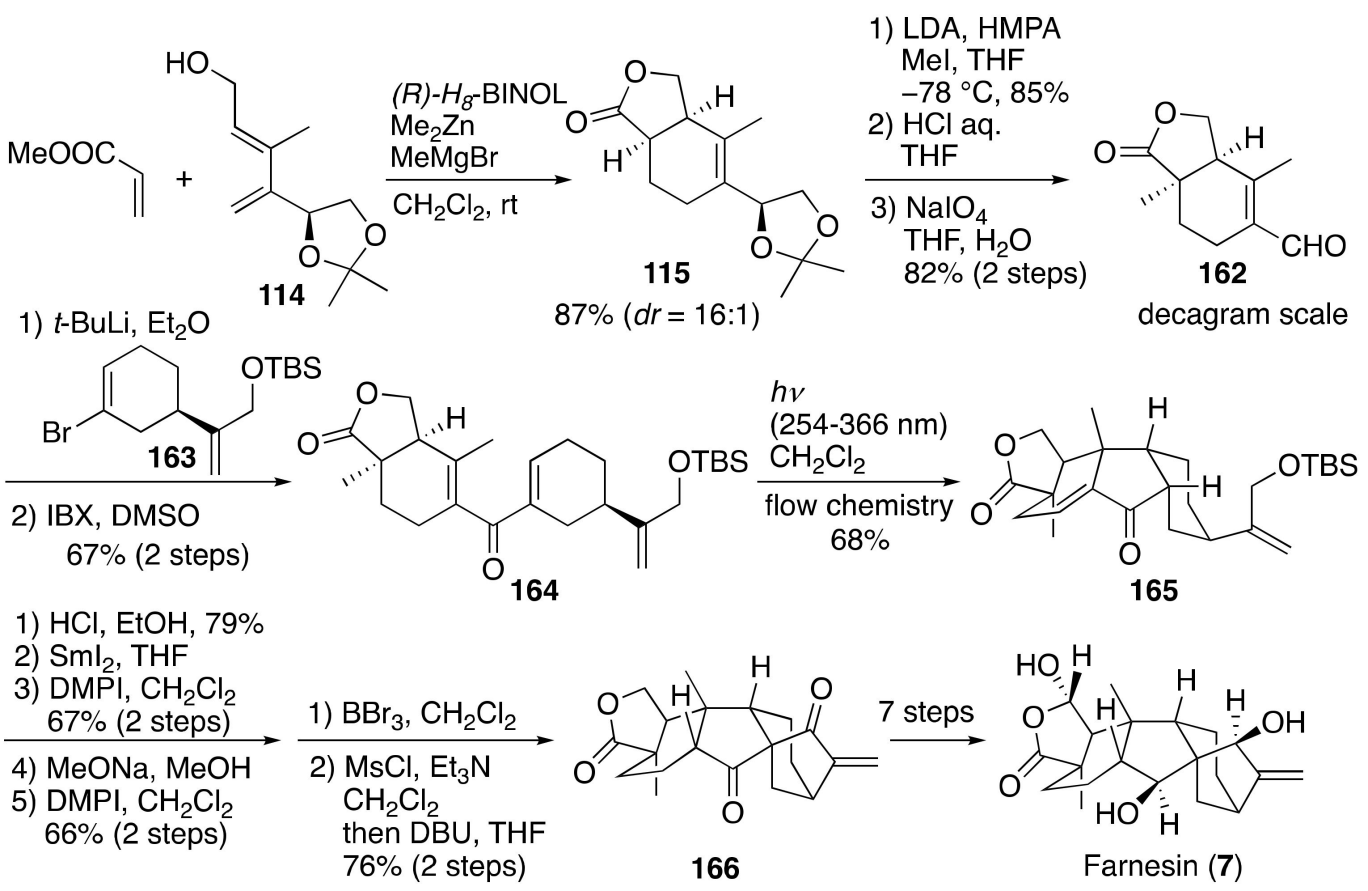
Gao’s total synthesis of farnesin.

In 2020, Gao et al. reported the total synthesis of farnesin involving photo-Nazarov and intramolecular aldol cyclization (Scheme 34) [40]. Their synthesis started with the construction of bicyclic 𝛾-lactone **114** via Ishihara’s Lewis-acid-template-catalyzed Diels–Alder reaction of **114** and methyl acrylate. Notably, the cycloaddition was scalable and afforded the desired adduct in a good yield with good diastereoselectivity. After α-methylation of **115**, the acetonide group was converted to aldehyde **162** via oxidative cleavage. The coupling of aldehyde **162** with compound **163** followed by oxidation afforded ketone **164**. Compound **164** was then subjected to a key photo-Nazarov reaction to ensure that the complex hydrofluorenone **165** was a single diastereomer. Compound **165** was converted to the pentacyclic product **166** via a SmI_2_-promoted radical reaction. From compound **166**, the total synthesis of farnesin was achieved via a seven-step transformation.

## 8. Conclusions

In this review, template-mediated Diels–Alder reactions that afford substituted bicyclic 𝛾-lactones were described. The Diels–Alder reactions shown here are useful and practical methods that facilitate access to valuable building blocks for the synthesis of complex natural products. The author hopes that this review will aid the development of novel strategies for the synthesis of complicated natural products and related substances.
